# Phylogenetic analysis of the true water bugs (Insecta: Hemiptera: Heteroptera: Nepomorpha): evidence from mitochondrial genomes

**DOI:** 10.1186/1471-2148-9-134

**Published:** 2009-06-15

**Authors:** Jimeng Hua, Ming Li, Pengzhi Dong, Ying Cui, Qiang Xie, Wenjun Bu

**Affiliations:** 1 Department of Zoology and Developmental Biology, Institute of Entomology, College of Life Sciences, Nankai University, Tianjin 300071, PR China; 2 Department of Agronomy, Tianjin Agricultural University, Tianjin 300071, PR China

## Abstract

**Background:**

The true water bugs are grouped in infraorder Nepomorpha (Insecta: Hemiptera: Heteroptera) and are of great economic importance. The phylogenetic relationships within Nepomorpha and the taxonomic hierarchies of Pleoidea and Aphelocheiroidea are uncertain. Most of the previous studies were based on morphological characters without algorithmic assessment. In the latest study, the molecular markers employed in phylogenetic analyses were partial sequences of 16S rDNA and 18S rDNA with a total length about 1 kb. Up to now, no mitochondrial genome of the true water bugs has been sequenced, which is one of the largest data sets that could be compared across animal taxa. In this study we analyzed the unresolved problems in Nepomorpha using evidence from mitochondrial genomes.

**Results:**

Nine mitochondrial genomes of Nepomorpha and five of other hemipterans were sequenced. These mitochondrial genomes contain the commonly found 37 genes without gene rearrangements. Based on the nucleotide sequences of mt-genomes, Pleoidea is not a member of the Nepomorpha and Aphelocheiroidea should be grouped back into Naucoroidea. Phylogenetic relationships among the superfamilies of Nepomorpha were resolved robustly.

**Conclusion:**

The mt-genome is an effective data source for resolving intraordinal phylogenetic problems at the superfamily level within Heteroptera. The mitochondrial genomes of the true water bugs are typical insect mt-genomes. Based on the nucleotide sequences of the mt-genomes, we propose the Pleoidea to be a separate heteropteran infraorder. The infraorder Nepomorpha consists of five superfamilies with the relationships (Corixoidea + ((Naucoroidea + Notonectoidea) + (Ochteroidea + Nepoidea))).

## Background

The true water bugs are grouped as the infraorder Nepomorpha, one of seven infraorders within the suborder Heteroptera (Insecta: Hemiptera) [1]. This group is of tremendous economic importance because all the members, except some Corixidae, are predators [2]. Extant water bugs have been grouped into 6 [3,4] or 5 superfamilies (Notonectoidea including Pleoidea) [2]. When molecular sequence data was combined with morphological characters to analyze the phylogenetic relationships of the true water bugs for the first time, a 7-superfamily classification system was proposed, containing a newly erected superfamily Aphelocheiroidea, which had formerly belonged to Naucoroidea (Table 1) [5].

**Figure 1:**
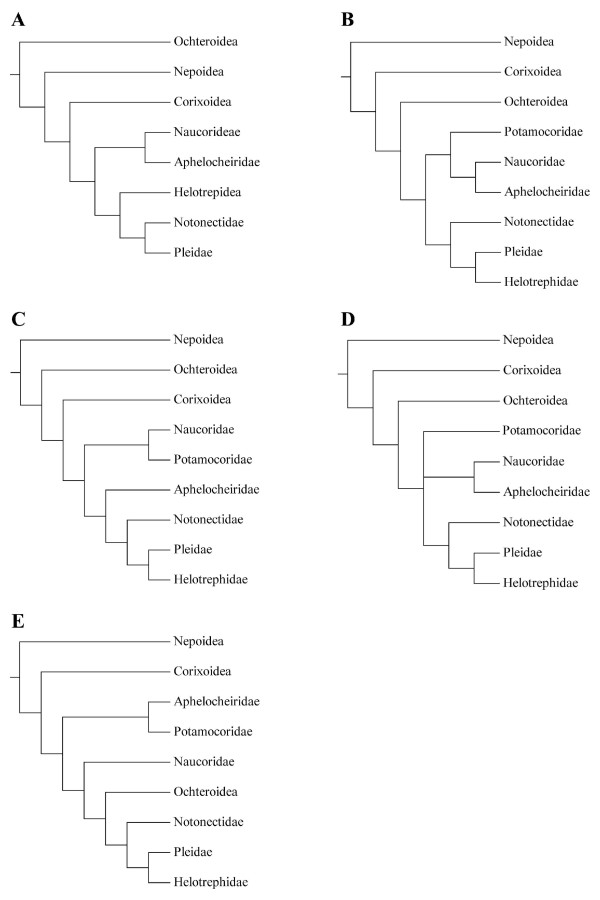
**Proposed phylogenetic hypotheses within Nepomorpha**. A, after China (1955); B, after Popov (1971); C, after Rieger (1976); D, after Mahner (1993); E, after Hebsgaard et al. (2004).

In these classification systems, the contents of Nepoidea, Corixoidea, and Ochteroidea are constant. Phylogenetic relationships proposed previously at the superfamily or family level within Nepomorpha are summarized in Figure 1. The works of China (1955) [6], Popov (1971) [7], Rieger (1976) [8], and Mahner (1993) [3] were based on morphological characters without algorithmic analysis. In their landmark work, Hebsgaard et al. (2004) reviewed these studies in detail and analyzed the phylogeny of Nepomorpha using molecular sequence data (partial sequence of 16S rDNA and 28S rDNA) combined with morphological data for the first time, raising a new superfamily Aphelocheiroidea as part of a new phylogenetic hypothesis (Figure 1E) [5].

Until now, the monophyly of each nepomorphan family, and the monophyly of superfamilies Nepoidea, Corixoidea, and Ochteroidea in Nepomorpha, have been generally accepted [3,5-8]. A close relationship between Pleidae and Helotrephidae has been supported by recent studies [3,5,7,8] and the monophyly of Aphelocheiridae + Potamocoridae was supported in the latest comprehensive study [5]. The phylogenetic relationships within Nepomorpha, however, have not reached full agreement. The unsolved problems are: 1) whether the Aphelocheiridae and Potamocoridae should be members of Naucoroidea or be raised as a separate superfamily Aphelocheiroidea; 2). whether the Pleidae and Helotrephidae should be members of Notonectoidea or be raised as a separate superfamily Pleoidea; 3) the phylogenetic relationships among the superfamilies.

The mitochondrial genome (mt-genome) is one of the largest sets of homologous genes which can be compared across animal taxa and has become an effective data source for resolving deep-level phylogenetic problems [9,10]. Within Insecta, more than one hundred mt-genomes are available now in GenBank/DDBJ/EMBL and mt-genomes have been shown to resolve intraordinal relationships, such as in Diptera [11], Hymenoptera [12], and Orthoptera [13]. There are many possible ways of using mt-genomes in phylogenetic analyses, for example by using different genes, amino acid sequences or nucleotide sequences. Using the nucleotide sequences of all available genes clearly has been shown to be the best way to extract a phylogenetic signal from mt-genomes [11,13].

**Table 1:** Previous classification systems of Nepomorpha

	**Štys and Jansson 1988; Mahner 1993**	**Schuh and Slater 1995**	**Hebsgaard et al. 2004**
Nepoidea	Belostomatidae	Belostomatidae	Belostomatidae
	Nepidae	Nepidae	Nepidae
Corixoidea	Corixidae	Corixidae	Corixidae
Ochteroidea	Ochteridae	Ochteridae	Ochteridae
	Gelastocoridae	Gelastocoridae	Gelastocoridae
Notonectoidea	Notonectidae	Notonectidae	Notonectidae
		Pleidae	
		Helotrephidae	
Pleoidea	Pleidae		Pleidae
	Helotrephidae	-	Helotrephidae
Naucoridae	Naucoridae	Naucoridae	Naucoridae
	Potamocoridae	Potamocoridae	
	Aphelocheiridae	Aphelocheiridae	
Aphelocheiridae	-	-	Potamocoridae
			Aphelocheiridae

Previous molecular data used for the analysis of nepomorphan relationships was about 1 kb [5] and no mt-genome data are available for any nepomorphan species. In this study, fourteen new mt-genomes were sequenced, nine of them belonging to the infraorder Nepomorpha (Table 2) (one mt-genome from our previous paper was also included in Table 2[14]). A preliminary phylogenetic framework of Nepomorpha is proposed using mt-genome data, and the relationships of Pleoidea and Aphelocheiroidea are analyzed.

**Table 2:** General informatics of the taxa used in this study

Classification	Species	Mt-genome Completeness	Size (bp)	A+T %	Accession Number
Archaeorrhyncha					
Fulgoroidea					
Fulgoridae	*Lycorma delicatula*	complete	15410	76.3	FJ456942
Heteroptera					
Gerromorpha					
Hydrometroidea					
Hydrometridae	*Hydrometra *sp.	complete	15416	78.7	FJ456945
Gerroidea					
Gerridae	*Gerris *sp.	complete	15380	75.7	FJ456944
Leptopodomorpha					
Leptopodoidea					
Leptopodidae	*Leptopus *sp.	*tRNA(I) – 12S*	14516	72.4	FJ456946
Cimicomorpha					
Reduvioidea					
Reduviidae	*Valentia hoffmanni*	complete	15625	73.7	FJ456952
Pentatomomorpha					
Coreoidea					
Rhopalidae	*Stictopleurus subviridis*	complete	15319	75.7	EU826088*
Nepomorpha					
Ochteroidea					
Gelastocoridae	*Nerthra *sp.	complete	16079	74.2	FJ456943
Ochteridae	*Ochterus marginatus*	*tRNA(I) – 12S*	14609	72.7	FJ456950
Corixoidea					
Corixidae	*Sigara septemlineata*	complete	15724	75.2	FJ456941
Notonectoidea					
Notonectidae	*Enithares tibialis*	complete	15262	76.1	FJ456949
Nepoidea					
Belostomatidae	*Diplonychus rusticus*	*tRNA(I) – 12S*	14596	69.8	FJ456940
Nepidae	*Laccotrephes robustus*	complete	15321	70.6	FJ456948
Naucoroidea					
Naucoridae	*Ilyocoris cimicoides*	complete	15209	71.0	FJ456947
Aphelocheiroidea					
Aphelocheiridae	*Aphelocheirus ellipsoideus*	*tRNA(I) – 12S*	14574	74.7	FJ456939
Pleoidea					
Pleidae	*Paraplea frontalis*	complete	15130	76.5	FJ456951

* From Hua et al. (2009) [14].

## Results

### General features of the fourteen mt-genomes of Hemiptera

Ten complete and four nearly complete mt-genomes of Hemiptera were sequenced (Table 2). Because of the polynucleotide regions, the control regions of the four incomplete mt-genomes were difficult to sequence. All mt-genomes are similar to the typical insect mt-genomes, containing the commonly found 37 genes in the same gene order as observed in *Drosophila yakuba *[GenBank: NC001322] [15]. No gene rearrangement or duplication has been found, indicating that the organizations of the mt-genomes in the suborder Heteroptera are more stable than those in the suborder Sternorrhyncha (Insecta: Hemiptera) [16]. Family names are used instead of the species names in following discussion for brevity since only a single representative was selected for each family.

Ten completely sequenced mt-genomes range in length from 15130 bp (Pleidae) to 16079 bp (Gelastocoridae). Lengths of the 37 coding genes of each mt-genome range from 14372 bp (Fulgoridae) to 14637 bp (Notonectidae), while the size of the control regions ranges from 608 bp (Pleidae) to 1450 bp (Gelastocoridae) (Table 3). Variance of the mt-genome size is mainly derived from the control region.

Nucleotide compositions of these mt-genomes are AT-biased (Table 3). Though the control regions of four mt-genomes were not completely sequenced, this biased trend is obvious even without the control region sequence. Interestingly, in most of the completely sequenced mt-genomes, the control regions are not the most AT-rich regions (Table 3), which has been found in other insect mt-genomes [15,17]

Intergenic spacers ranging from 14 bp (Corixidae) to 23 bp (Reduviidae) were found between *tRNA-Ser(UCN) *and *ND1 *in all mt-genomes except Fulgoridae. This spacer has also been reported in other insects and some conserved motifs were identified [18-20]. In the present study, no perfectly conserved motif was found but a conserved region was identified (see additional file 1). Additionally, a unique 222 bp intergenic spacer was found between *tRNA-Gly *and *ND3 *in Hydrometridae which lacks significant BLAST similarity (megablast in nucleotide collection). The exact origin and function of such spacers are unclear, but they may be the vestiges of pseudogenes generated by the gene duplication-random loss process of rearrangement [21]

Other features of these mt-genomes, including non-traditional start codons such as TTG of *CO1 *in Naucoridae and GTG of ND1 in Aphelocheiridae, incomplete stop codons T or TA, and absence of the DHU arm in the secondary structure of tRNA-Ser(GCU), are found commonly in insect mt-genomes [15,18,20,22-24].

### Phylogenetic analyses

**Figure 2:**
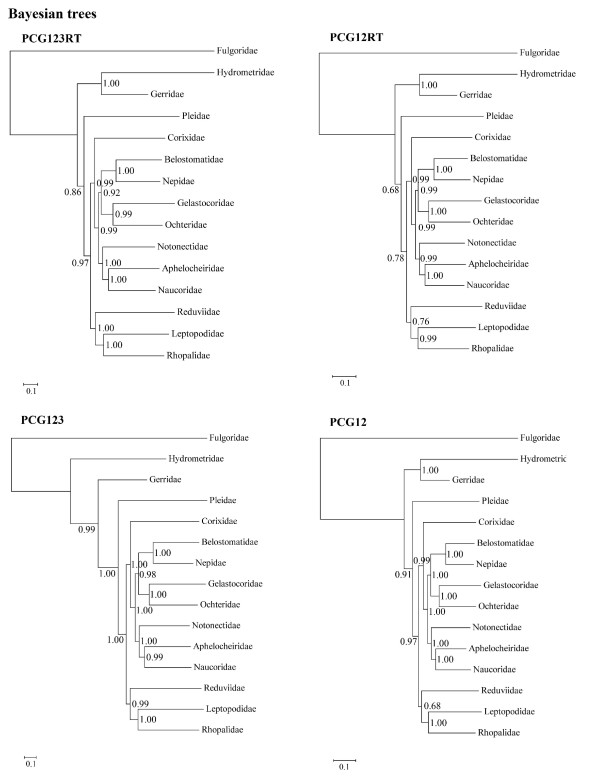
**Bayesian phylograms inferred from PCG123RT, PCG12, PCG123, and PCG12 data sets**. Bayesian posterior probabilities are indicated at each node.

**Figure 3:**
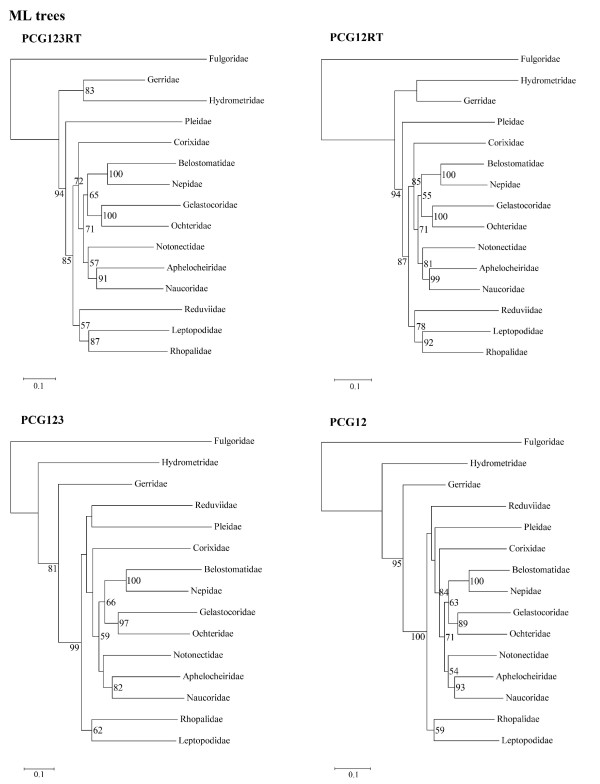
**ML phylograms inferred from PCG123RT, PCG12, PCG123, and PCG12 data sets**. Bootstrap support values are indicated at each node.

For the fifteen species, there are 14968 sites in the PCG123RT matrix (containing all three codon positions for protein coding genes (PCGs), plus the whole of the rRNA and tRNA genes), 11236 sites in the PCG12RT matrix (containing the first and the second codon positions of PCGs, plus the whole of the rRNA and tRNA genes), 11196 sites in the PCG123 matrix (containing all three codon positions of PCGs), and 7464 sites in the PCG12 matrix (containing the first and the second codon positions of PCGs). From Bayesian and ML inferences, these four matrices generated eight fully bifurcated trees with similar topology (Figure 2, Figure 3). The monophyletic Nepoidea and Ochteroidea were consistently recovered. The monophyly of the remaining nepomorphan superfamilies such as Pleoidea, Corixoidea, Notonectoidea, Naucoroidea, and Aphelocheiroidea could not be analyzed in this study as only a single representative was sampled. In all trees, the relationships within Nepomorpha were found to be constant. The Pleoidea (Pleidae) was recognized as the sister group of the clade including the infraorders Cimicomorpha (Reduviidae), Leptopodomorpha (Leptopodidae), Pentatomomorpha (Rhopalidae), and the remaining traditional Nepomorpha. Nepoidea and Ochteroidea were sister groups and this clade was found to be a sister group of Notonectoidea (Notonectidae) plus Naucoroidea (Naucoridae and Aphelocheiridae).

**Table 3:** Statistics of the length and nucleotide composition of the genes

Taxa	Lengths of total genes	AT% of total genes	Lengths of control region	AT% of control region
Fulgoridae	14372	75.8	1043	83.3
Hydrometridae	14478	78.6	694	78.1
Gerridae	14616	73.1	781	66.2
Leptopodidae	14532	72.4	-	-
Reduviidae	14570	73.8	724	69.9
Rhopalidae	14563	75.6	685	77.1
Gelastocoridae	14631	75.1	1450	65.4
Ochteridae	14601	72.7	-	-
Corixidae	14504	75.4	772	76.7
Notonectidae	14637	76.3	646	70.4
Belostomatidae	14573	69.8	-	-
Nepidae	14575	70.9	751	65.8
Naucoridae	14611	71.2	609	66.0
Aphelocheiridae	14582	74.8	-	-
Pleidae	14559	76.5	608	75.0

## Discussion

### Mitochondrial genomes

The mt-genomes sequenced in this study are similar to the mt-genomes of other insects and contain very few novel features (see additional file 2 for the descriptions of these mt-genomes). It has been reported that the hemipteroid insects (including Hemiptera, Thysanoptera, Psocoptera, and Phthiraptera) have experienced increased rates of mt-genomic gene rearrangements [16,25-29]. Gene content and gene order of the mt-genomes sequenced in this study, however, are all the same as observed in *Drosophila yakuba *[GenBank: NC001322) [15] except for some unique intergenic spacers. In the present study, gene order provides no phylogenetic information.

### Phylogenetic analyses

Mt-genomes provide abundant phylogenetic signal because they possess large sets of homologous genes. Multiple genes with increased sequence lengths are favorable for accurate phylogenetic analyses [30-32]. It has been shown that the best way to use mt-genomes in phylogenetic analyses is to combine all the coding genes and use nucleotide sequence data [13]. We used a data set consisting of all 37 genes (PCG123RT data set) to infer relationships within Nepomorpha. The PCG12RT, PCG123, and PCG12 data sets were analyzed to test the stability of the phylogenetic hypotheses to the inclusion of different portions of the data. Phylogenetic relationships among heteropteran infraorders are still controversial [33-36]. Taxon sampling for this study is too limited to analyze this problem in full and data for the infraorders Enicocephalomorpha and Dipsocoromorpha are not yet available. We focused on the phylogeny within infraorder Nepomorpha, especially relationships among superfamilies, using mt-genome sequences for the first time. Finally, five of the eight analyses inferred the same topology, with the remaining three tree resulting form less complete datasets (nucleotide substitution saturation analysis was also added in additional file 3) differing at a few nodes, which indicates that the mt-genome is an effective data source for resolving phylogenetic problems within Nepomorpha at the superfamily level. Because the ML and Bayesian algorithms are not sensitive to possible long-branch attraction and allow evolutionary modeling of the data [37-39], we do not think our hypotheses are artifacts.

### The Problem of Pleoidea

The Pleoidea has been proposed to include Pleidae and Helotrephidae [3,5]. The mt-genome of Helotrephidae was not sequenced in this study. This family has been generally accepted as the sister group of Pleidae [3,5,7,8] except in the study of China (1955) [6].

Previous studies based on morphological characters, molecular data or combined data consistently support a monophyletic Nepomorpha [3,5-8]. In this study, however, a monophyletic Nepomorpha is supported by one analysis of the eight performed. In all previous studies, Pleoidea is the sister group of Notonectoidea [3,5,7,8] and has always been included in Notonectoidea [2]. A surprising, but strongly supported result from the mt-genome analysis was that Pleoidea is not part of Nepomorpha, but rather the sister group of the clade including Nepomorpha, Leptopodomorpha, Cimicomorpha, and Pentatomomorpha (Figure 2, Figure 3).

Based on these results we propose that the Pleoidea could be raised from a superfamily to the infraorder Plemorpha, and that the infraorder Nepomorpha consists of the remaining nepomorphans except Pleoidea. The phylogenetic position of the infraorder Plemorpha within suborder Heteroptera needs further study because mt-genome data from infraorders Enicocephalomorpha and Dipsocoromorpha are unavailable at the present time.

### The Problem of Aphelocheiroidea

The recently proposed superfamily Aphelocheiroidea (including Aphelocheiridae and Potamocoridae) [5], which was considered as part of Naucoroidea by other researchers [2,3], was proposed to be the sister group of a clade consisting of Ochteroidea, Notonectoidea, and Naucoroidea [5]. In the present analysis, Aphelocheiridae and Naucoridae consistently formed a monophyletic clade with high data support (Figure 2, Figure 3). Although the mt-genome of Potamocoridae was not sequenced in this study, this family has been suggested to be the sister group of Aphelocheiridae [5], Naucoridae [8], Naucoridae + Aphelocheiridae [7], a subfamily within Naucoridae [6], or unresolved by Mahner (1993) [3]. Here we propose that the Aphelocheiroidea raised by Hebsgaard et al. (2004) [5] should be grouped within Naucoroidea again.

### Phylogeny of "Nepomorpha"

The traditional infraorder Nepomorpha is not monophyletic and it should only contain Corixoidea, Nepoidea, Ochteroidea, Notonectoidea, and Naucoroidea. The superfamilies Nepoidea (including Nepidae and Belostomatidae) and Ochteroidea (including Ochteridae and Gelastocoridae) are monophyletic and sister-groups in all trees with high support, as is generally accepted [3,5-8]. Nepoidea has been considered as the most basal branch of Nepomorpha in previous studies [3,5,7,8]. The position of Ochteroidea varied between previous researchers [3,5-8]. Because of the novel position inferred for Pleoidea, the sister-group relationship between Pleoidea and Notonectoidea which has been consistently proposed [3,5-8] was not supported and Notonectoidea was inferred to be the sister group of Naucoroidea.

The placement of Corixoidea is very different from previous hypotheses. Traditionally, this superfamily was placed as the sister group of a clade composed of Naucoroidea, Ochteroidea, and Notonectoidea [3,5,7] or the sister group of a clade composed of Naucoroidea and Notonectoidea [6,8]. In our results, Corixoidea is always the most basal clade within Nepomorpha, with Naucoroidea, Notonectoidea, Ochteroidea, and Nepoidea forming a monophyletic group.

Furthermore, *Lycorma delicatula *(Insecta: Hemiptera: Archaeorrhyncha) was removed from the data sets and the phylogeny was re-analyzed with the same methods. Same position of Pleoidea and phylogenetic hypotheses within Nepomorpha could be drawn (see additional file 4). Finally, the infraorder Nepomorpha should consist of five superfamilies with the phylogenetic hypothesis of (Corixoidea + ((Naucoroidea + Notonectoidea) + (Ochteroidea + Nepoidea))).

## Conclusion

Although previous studies based on morphological characters alone or combined with DNA sequence no longer than 1 kb confirmed the monophyly of Nepomorpha, the phylogenetic inference with the evidence from mitochondrial genomes in this study supports the raise of a separate infraorder Plemorpha which belonged to Nepomorpha before. The well-resolved nepomorphan phylogenetic relationships at superfamily level allow a better understanding of evolutionary patterns within this group and provide a robust framework for comparative studies of nepomorphans. The present study demonstrates the great effectiveness of mitochondrial genome for inferring phylogenetic relationships at superfamily level. Furthermore, this study also suggests the need of using multiple genes for future phylogenetic analyses of highly debated phylogenies.

## Methods

According to the seven-superfamily system of Nepomorpha proposed by Hebsgaard et al. (2004) [5], the representatives of each superfamily were selected (Table 2). Representatives of the infraorders Gerromorpha, Leptopodomorpha, Cimicomorpha and Pentatomomorpha were also included (Table 2). A representative of the suborder Archaeorrhyncha (Insecta: Hemiptera), *Lycorma delicatula *(White), was chosen to root the trees.

A single individual of each species was preserved in 95% ethanol at -20°C and total genomic DNA was extracted using the method based on CTAB [40]. PCRs were performed with TaKaRa LA PCR Kit Ver.2.1 following the manufacturer's recommendations. The primers are listed in additional file 5. PCR products were electrophoresed in 0.7% agarose gel, purified, and then both strands were sequenced with primer walking by Beijing Sunbiotech Co. Ltd.

The complete sequences of each gene were used for phylogenetic analysis (excluding stop codons of the PCGs). All PCGs were aligned based on amino acid sequence alignments in MEGA version 4.0 [41]. The *rRNAs *and the *tRNAs *were aligned with CLUSTAL X version 1.83 [42] under the default settings. Ambiguously aligned regions of PCGs and rRNA genes were carefully adjusted by hand. Transfer RNA alignments were corrected according to secondary structure. The aligned sequences were concatenated as four matrices used in phylogenetic analyses: 1) The PCG123RT matrix, including all three codon positions of PCGs, rRNA genes, and tRNA genes; 2) the PCG12RT matrix, including the first and the second codon positions of PCGs, rRNA genes, and tRNA genes; 3) the PCG123 matrix, including all the three codon positions of PCGs; 4) the PCG12 matrix, including the first and the second codon positions of PCGs.

MrBayes Version 3.1.1 [43] and a PHYML online web server [44] were employed to reconstruct the phylogenetic trees under the GTR model. In Bayesian inference, two simultaneous runs of 3,000,000 generations were conducted for each matrix. Trees inferred prior to stationarity were discarded as burn-in, and the remaining trees were used to construct a 50% majority-rule consensus tree. In ML analysis, the parameters were estimated during analysis and the node support values were assessed by bootstrap resampling (BP) [45] calculated using 100 replicates.

## Abbreviations

*CO1*, *CO2*, and *CO3*: were the abbreviations of cytochrome c oxidase subunit I, II, and III genes; *CytB*: cytochrome b gene; *ATP6 *and *ATP8*: ATP synthase F0 subunit 6 and 8 genes; *ND1*, *ND2*, *ND3*, *ND4*, *ND4L*, *ND5*, *ND6*: NADH dehydrogenase subunit 1–6 and 4L genes. Transfer RNA genes were labeled according to the IUPAC-IUB single letter code for the specified amino acid; in cases where there was more than one tRNA for a particular amino acid, they were distinguished by their anticodons. PCG: protein coding gene.

## Authors' contributions

JH designed the experiments, carried out the data analyses and drafted the manuscript. ML, PD, and YC participated in the experiments and helped prepare the additional files. QX helped draft the manuscript. WB directed this study and revised the manuscript. All authors read and approved the final manuscript.

## Supplementary Material

Additional file 1 **Alignments of the conserved sequence blocks of the intergenic spacers between *tRNA-S(TGA) *and *ND1***. The data provided represent the alignments of the conserved sequence blocks of the intergenic spacers between *tRNA-S(TGA) *and *ND1*. The alignments were generated by plotting the identities to a standard as a dot, and a gap as a dash. Click here for file

Additional file 2 **General information of the mt-genomes in this study**. The data provided represent the putative secondary structure of tRNAs, codon usage in each mt-genome, and analyses of nucleotide compositions of each mt-genome. Click here for file

Additional file 3 **Nucleotide substitution saturation analysis**. The data provided represent the nucleotide substitution saturation analysis of different portions of datasets. Click here for file

Additional file 4 **Bayesian and ML phylograms inferred from the data sets without *Lycorma delicatula *(Insecta: Hemiptera: Archaeorrhyncha)**. The data provided represent Bayesian and ML phylograms inferred from the data sets without *Lycorma delicatula *(Insecta: Hemiptera: Archaeorrhyncha). Click here for file

Additional file 5 **Primers used in PCR**. The data provided represent primers used in amplification of mt-genomes. Click here for file
