# Supplementary material for: Phylogenetic analysis of the true water bugs (Insecta: Hemiptera: Heteroptera: Nepomorpha): evidence from mitochondrial genomes
Source: BMC Evol Biol. 2009 Jun 15;9:134. doi: 10.1186/1471-2148-9-134 (PMC2711072; doi:10.1186/1471-2148-9-134)
Supplement: Additional file 1 — Alignments of the conserved sequence blocks of the intergenic spacers between tRNA-S(TGA) and ND1. The data provided represent the alignments of the conserved sequence blocks of the intergenic spacers between tRNA-S(TGA) and ND1. The alignments were generated by plotting the identities to a standard as a dot, and a gap as a dash. [file 1471-2148-9-134-S1.pdf]

Aphelocheiridae	CACAAAAAAAAATGA
Belostomatidae	. . . CT . . . .
Corixidae	. . . TT . TTT . . . . . -
Gelastocoridae	. . . TC . . . . . G . . . .
Gerridae	A . . TT . . . . TG . . . .
Hydrometridae	. . . T . . . TT . C . . . .
Leptopodidae	. . . T . . . TTT . . . . .
Naucoridae	. . . . G
Nepidae	A . . T . . . .
Notonectidae	. . . . TT . . . . .
Ochteridae	. . . .
Pleidae	. T . . . . TT . . . .
Reduviidae	A . . . TT . TTT . . AT .
Rhopalidae	. . . TT . . . . C . . . . .
