# Supplementary material for: Phylogenetic analysis of the true water bugs (Insecta: Hemiptera: Heteroptera: Nepomorpha): evidence from mitochondrial genomes
Source: BMC Evol Biol. 2009 Jun 15;9:134. doi: 10.1186/1471-2148-9-134 (PMC2711072; doi:10.1186/1471-2148-9-134)
Supplement: Additional file 2 — General information of the mt-genomes in this study. The data provided represent the putative secondary structure of tRNAs, codon usage in each mt-genome, and analyses of nucleotide compositions of each mt-genome. [file 1471-2148-9-134-S2.pdf]

Putative secondary structures of tRNAs

Based on the DNA sequences, most of the tRNAs could be folded as cloverleaf secondary structures. A few of them possessed non-Watson-Crick matches, aberrant loops, or even extremely short arms. Most of the tRNA-S (GCT) lost their DHU arms, which is not rare in insect mt-genomes. In some cases, the alternative folding could yield a structure with an extremely short DHU stem and a small loop. It has been supposed that these characteristics in mt-genome are partly because the mtDNA was not subject to the process of recombination, which may facilitate the elimination of deleterious mutations [1]. However, recombination in insect mtDNA has been observed [2]. It is not known whether the aberrant tRNAs lose their function in every case, but there are reports of the recruitment of nuclear tRNAs into the mitochondria [3, 4], and a type of RNA editing could recover the well-paired acceptor stem [5].

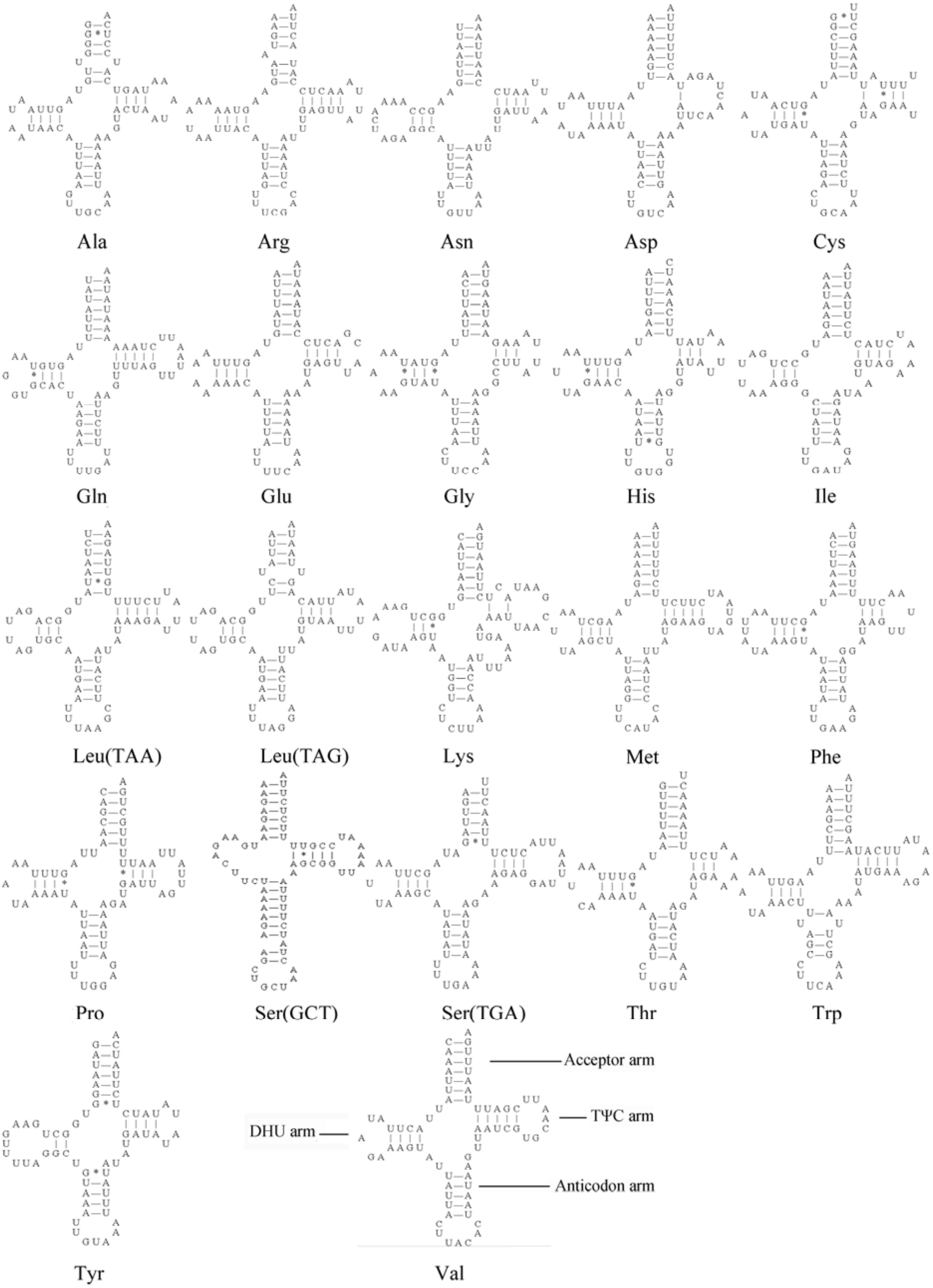

Figure 1 Putative secondary structures of mitochondrial tRNA molecules from Aphelocheiridae

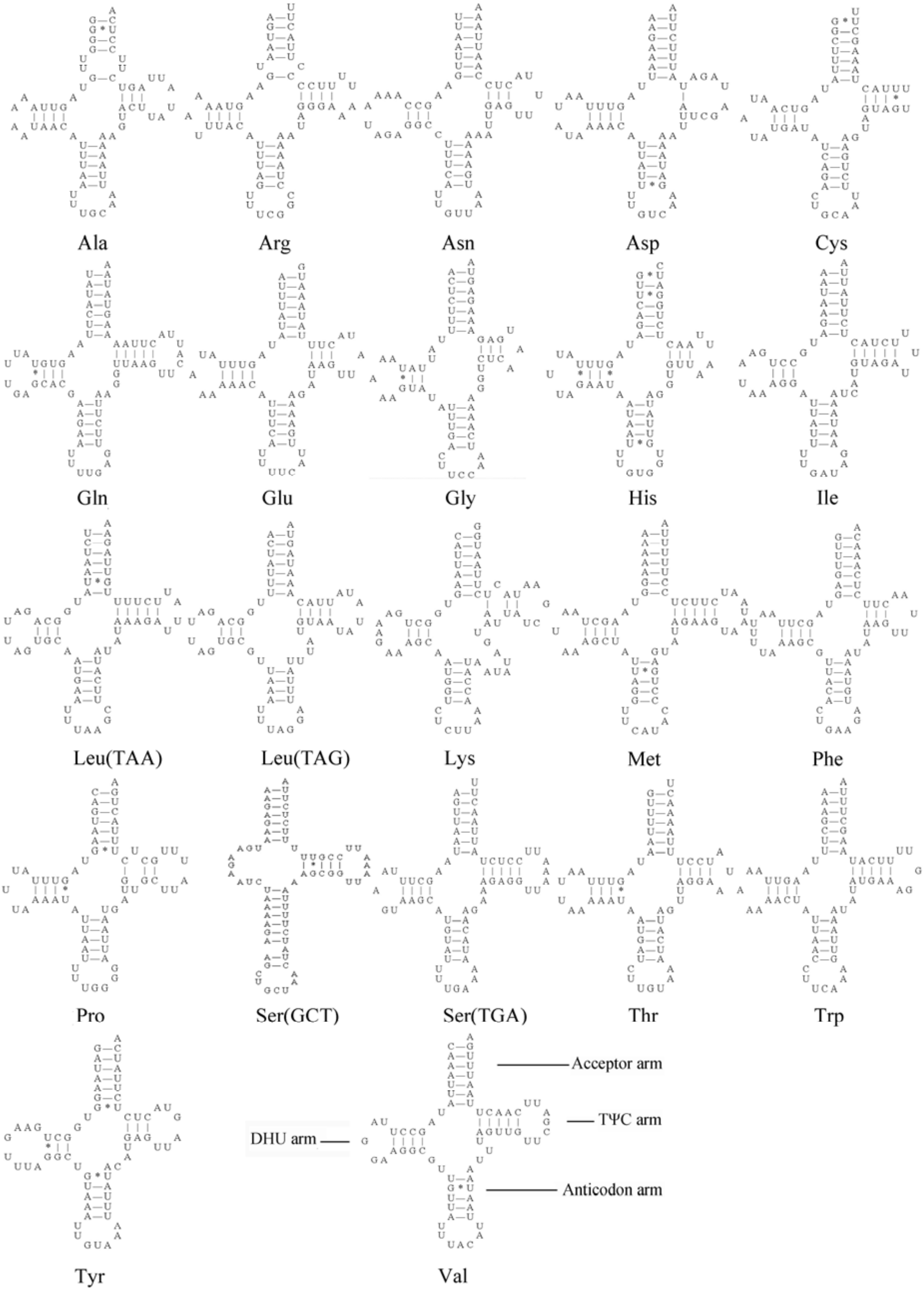

Figure 2 Putative secondary structures of mitochondrial tRNA molecules from Belostomatidae

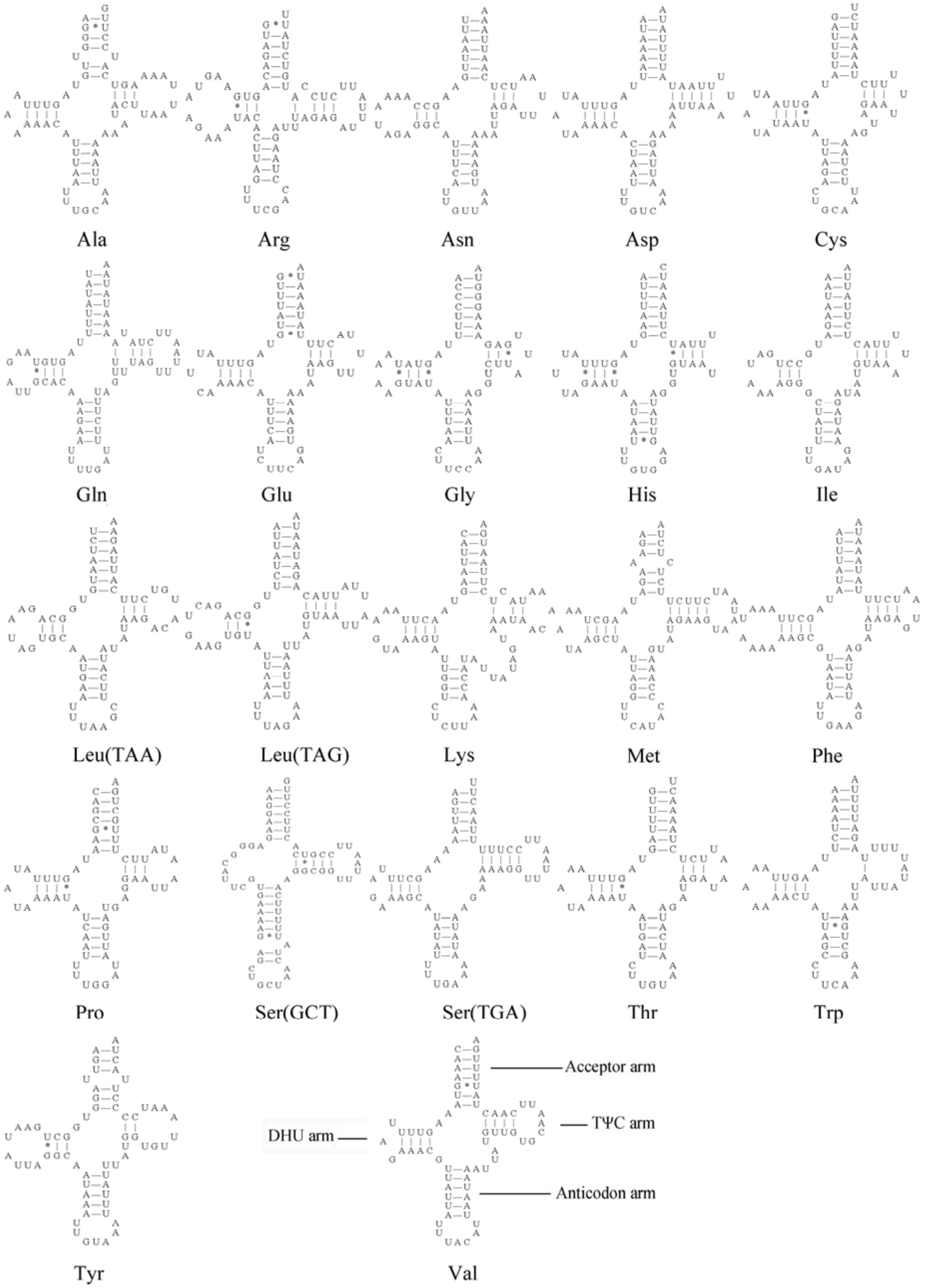

Figure 3 Putative secondary structures of mitochondrial tRNA molecules from Corixidae

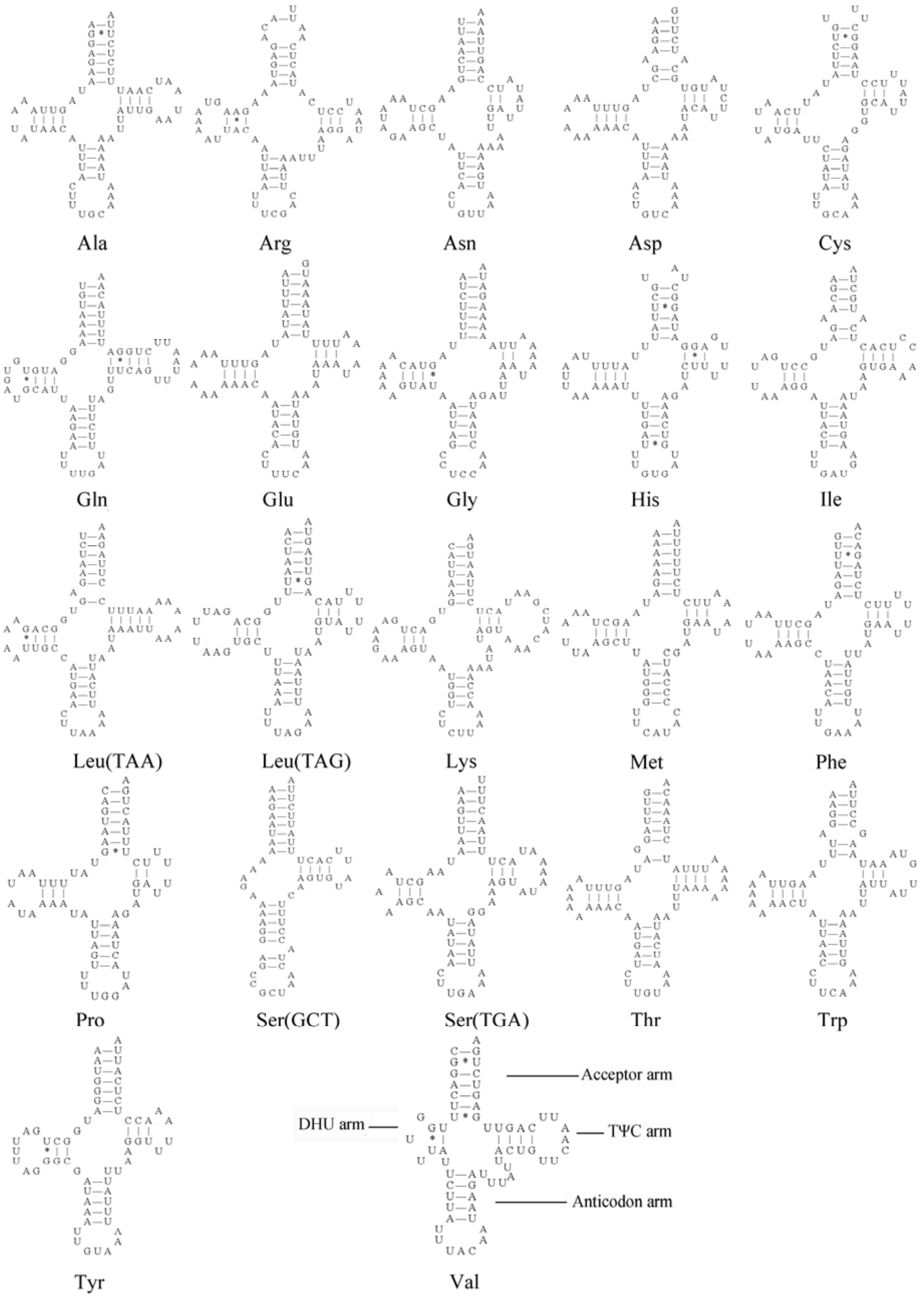

Figure 4 Putative secondary structures of mitochondrial tRNA molecules from Fulgoridae

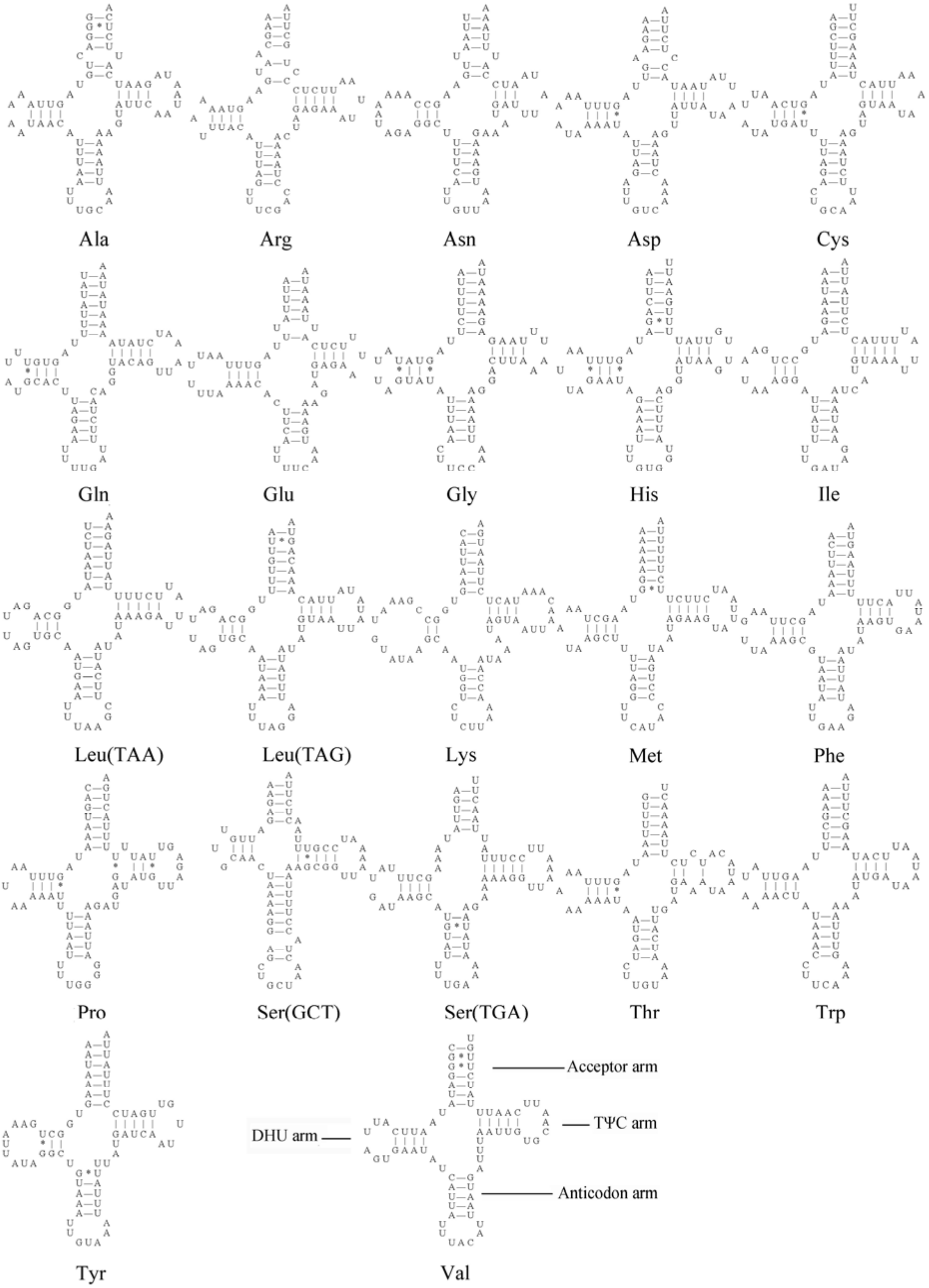

Figure 5 Putative secondary structures of mitochondrial tRNA molecules from Gelastocoridae

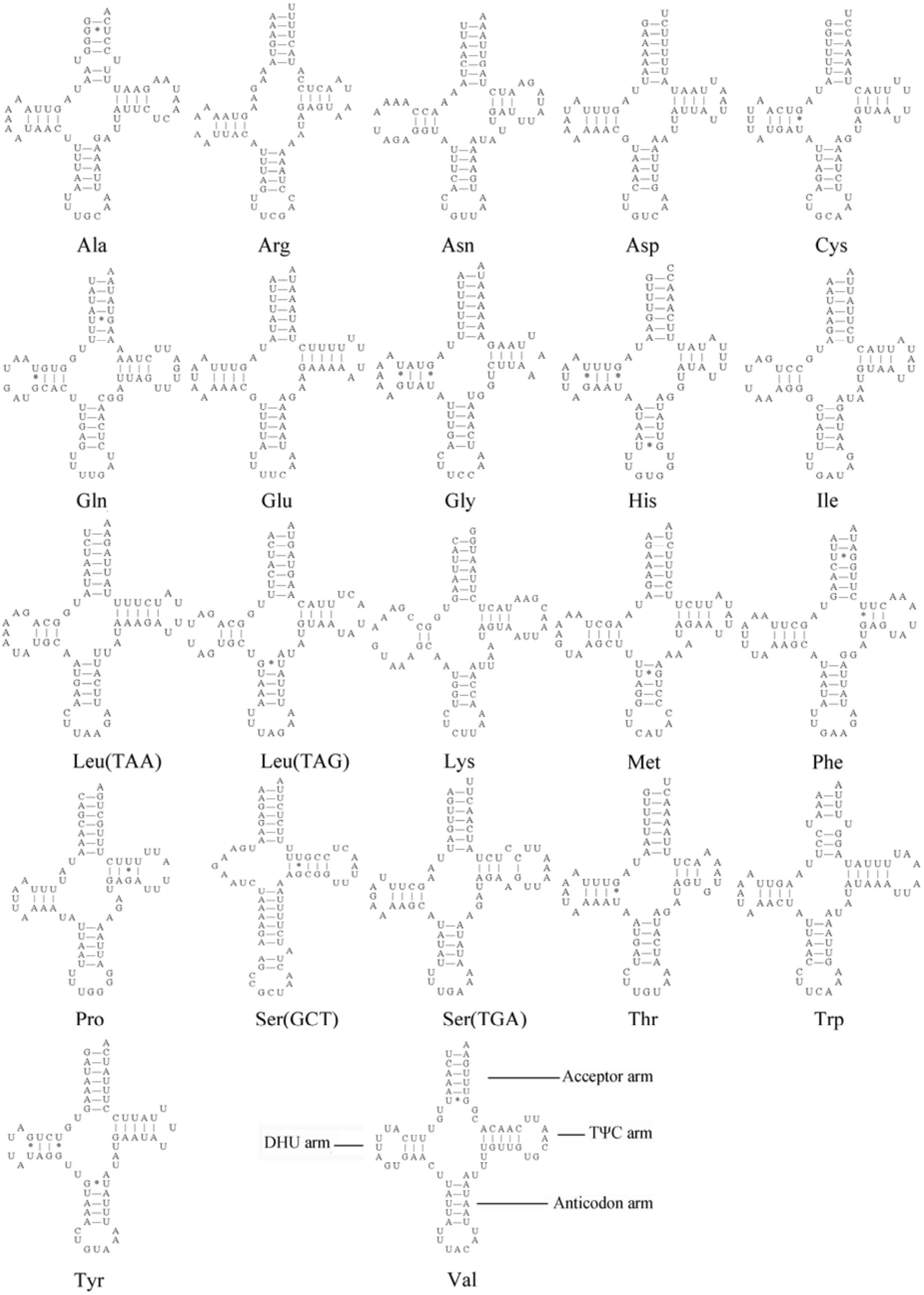

Figure 6 Putative secondary structures of mitochondrial tRNA molecules from Gerridae

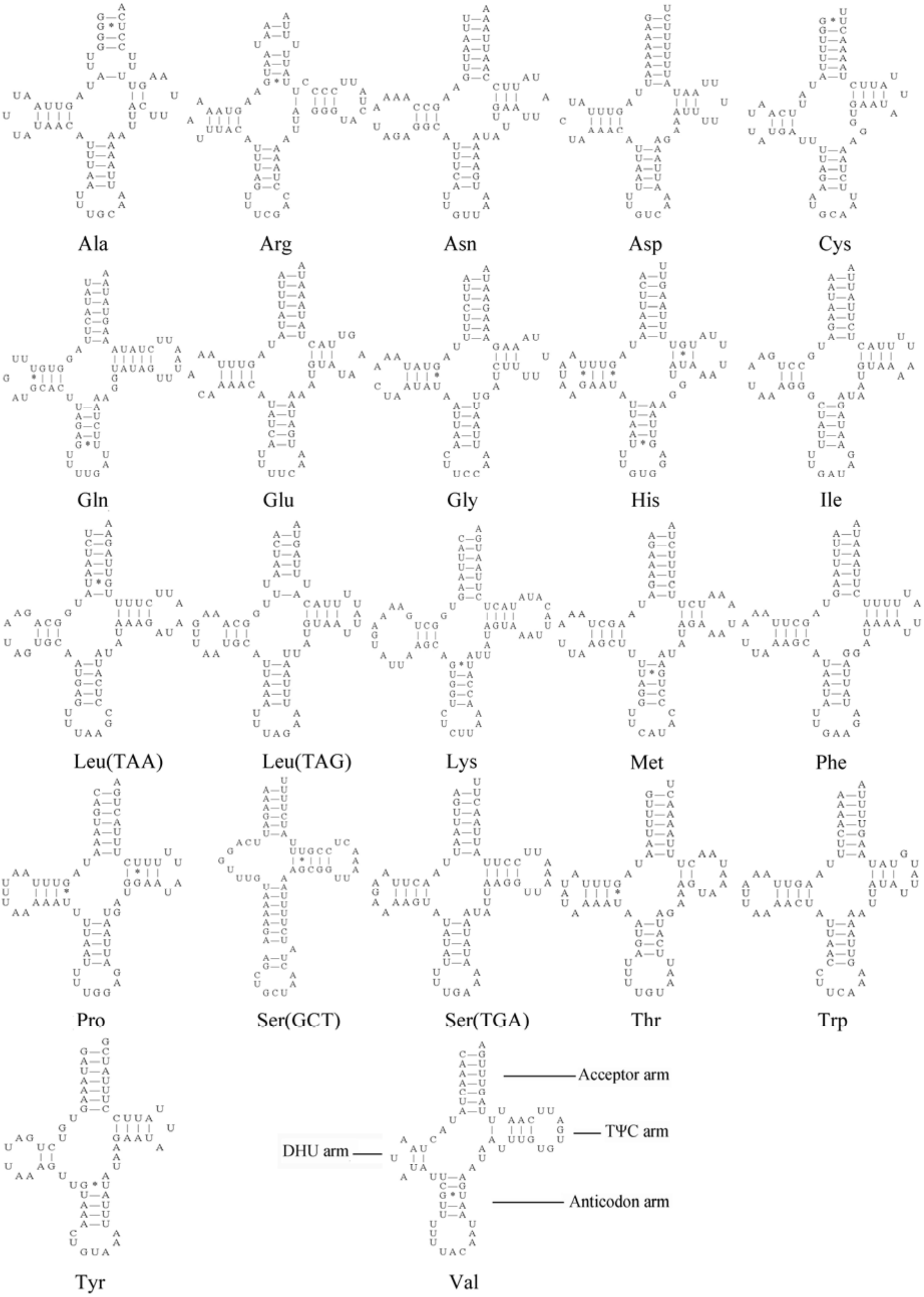

Figure 7 Putative secondary structures of mitochondrial tRNA molecules from Hydrometridae

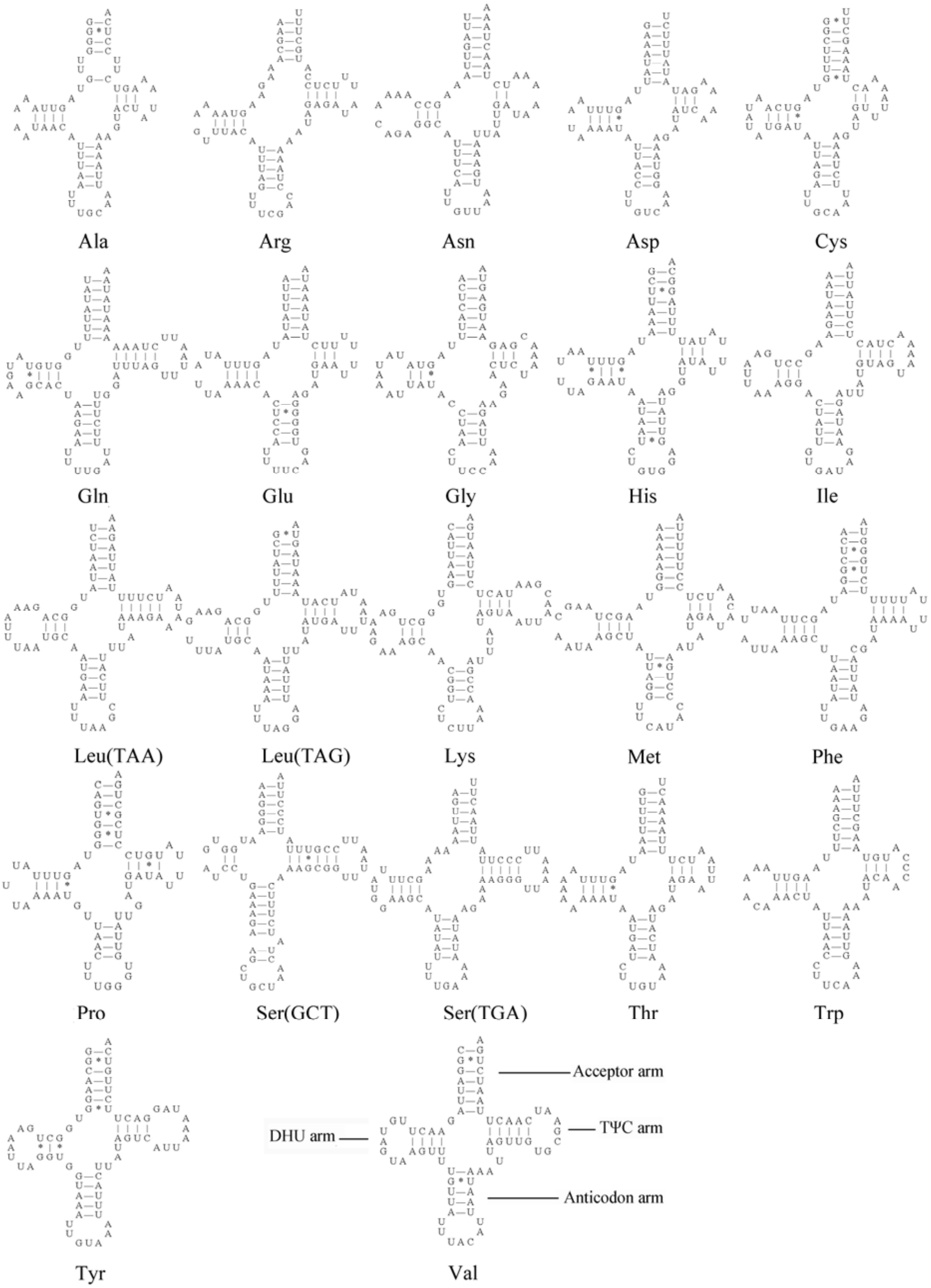

Figure 8 Putative secondary structures of mitochondrial tRNA molecules from Leptopodidae

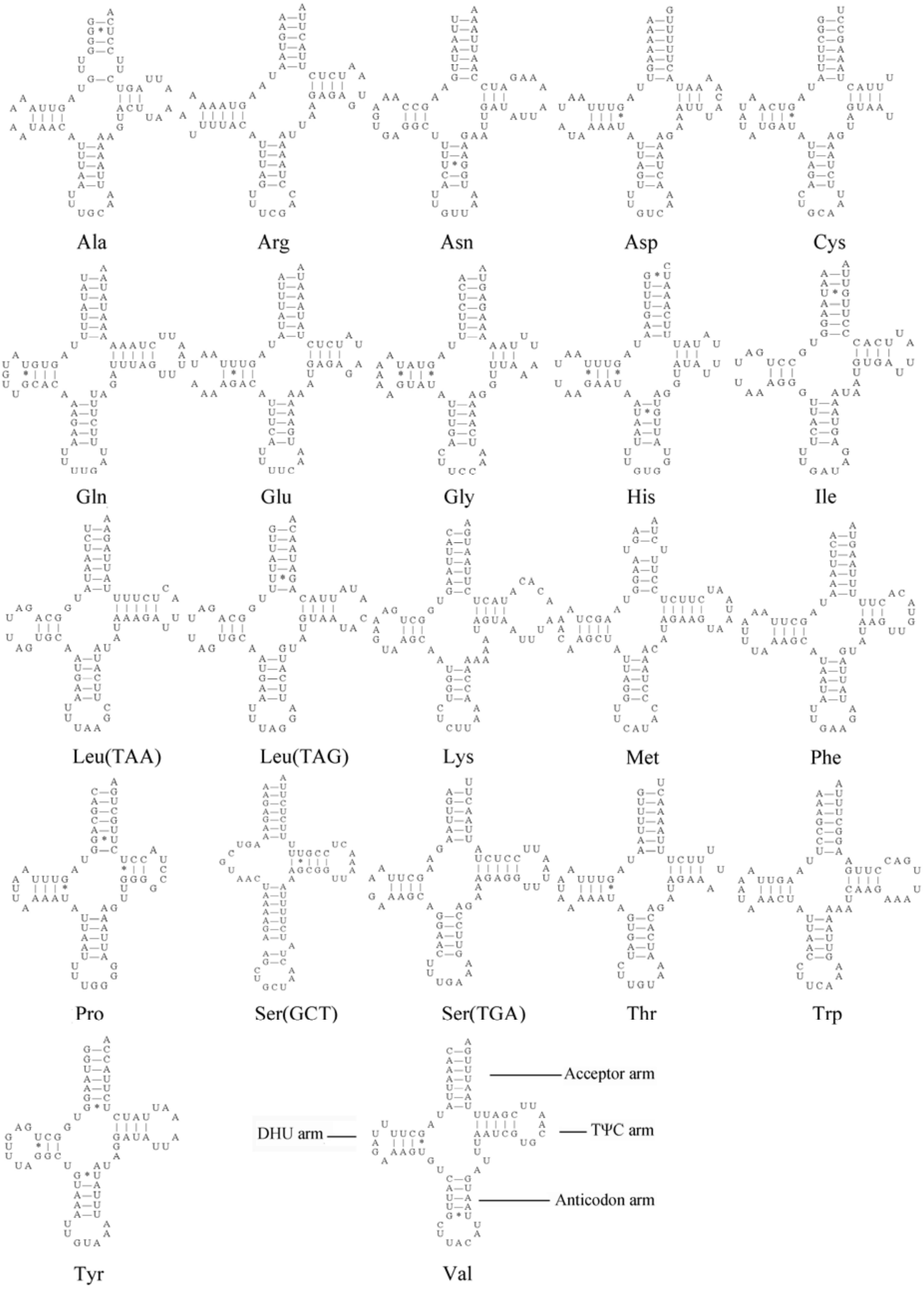

Figure 9 Putative secondary structures of mitochondrial tRNA molecules from Naucoridae

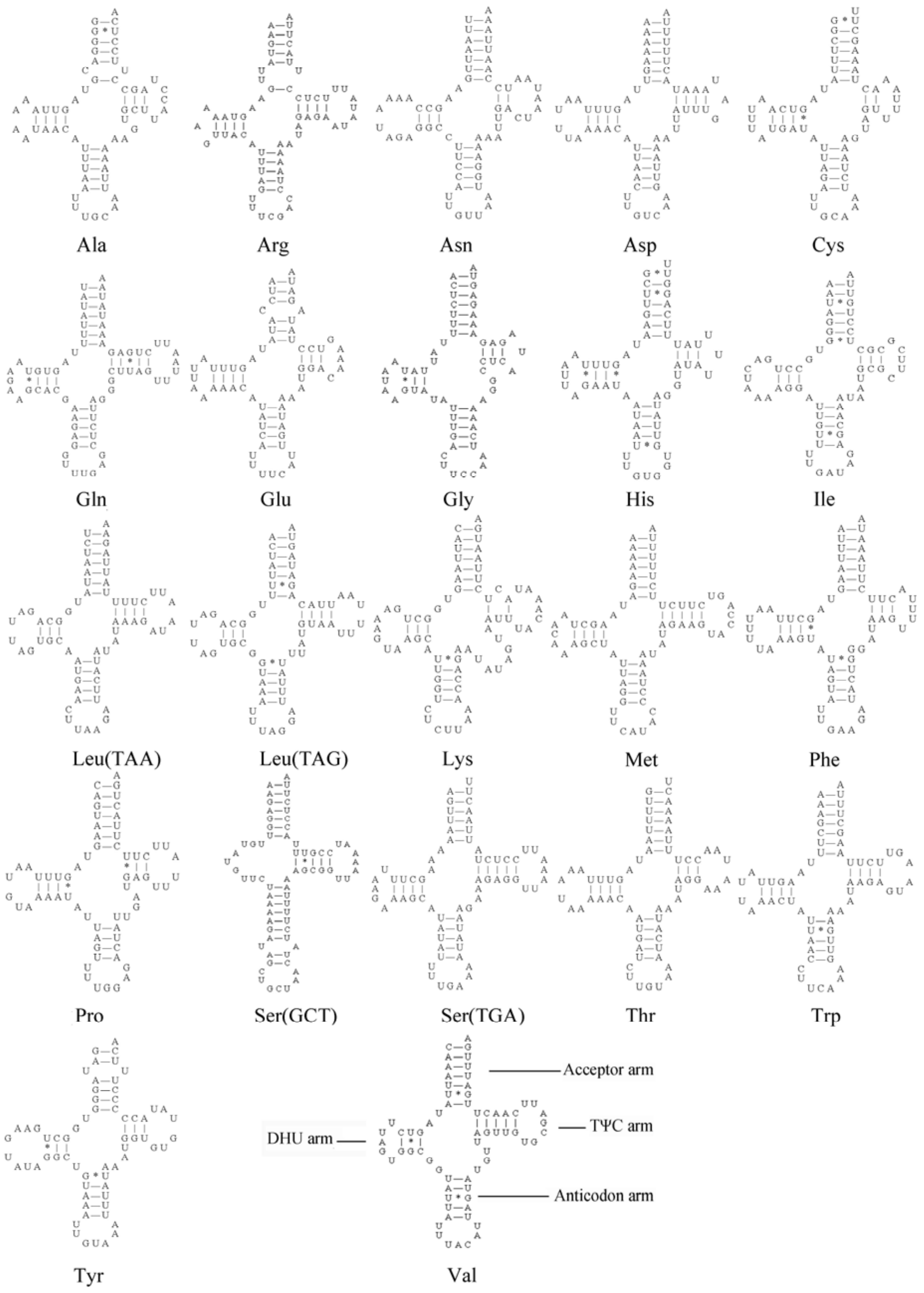

Figure 10 Putative secondary structures of mitochondrial tRNA molecules from Nepidae

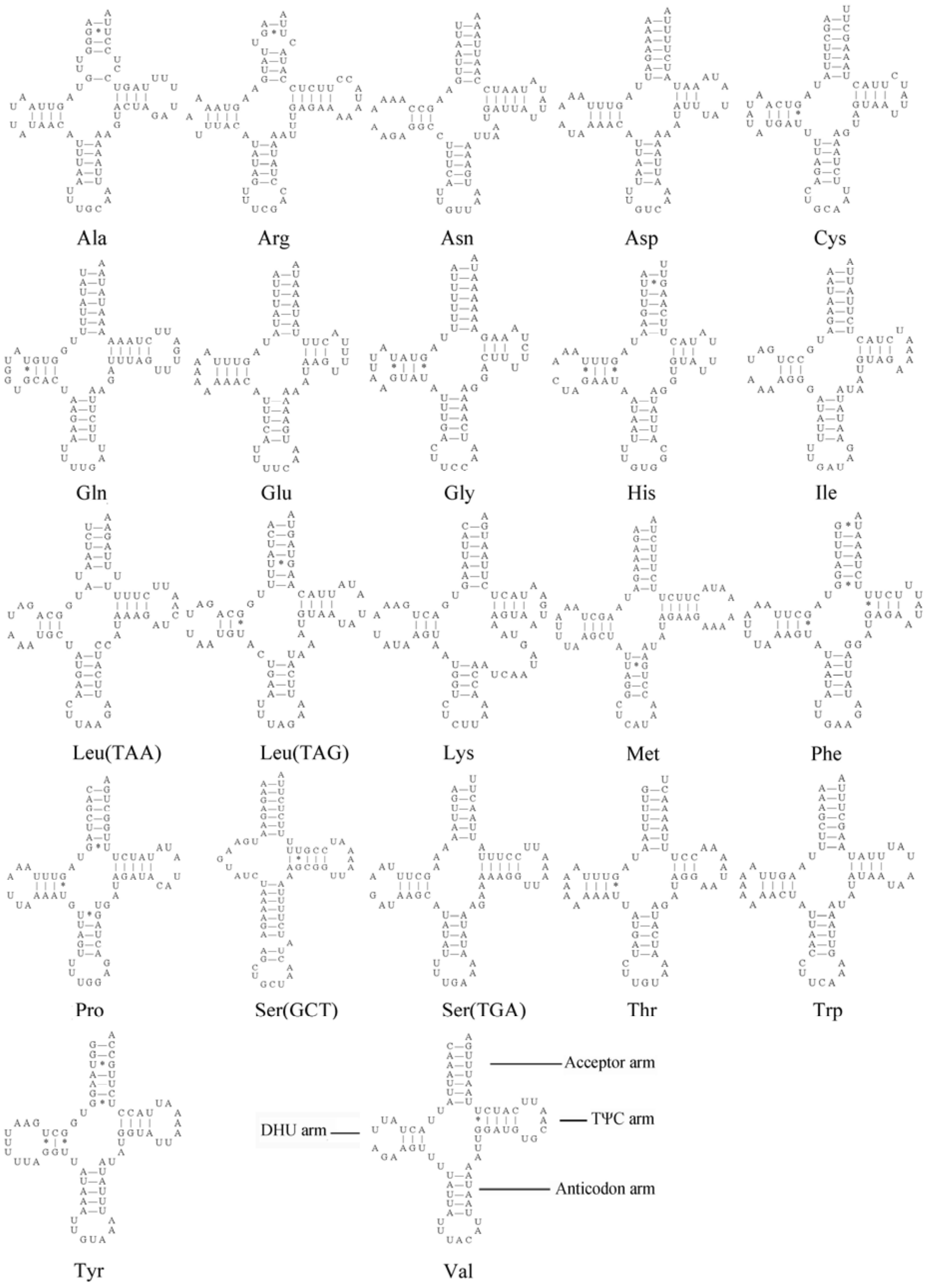

Figure 11 Putative secondary structures of mitochondrial tRNA molecules from Notonectidae

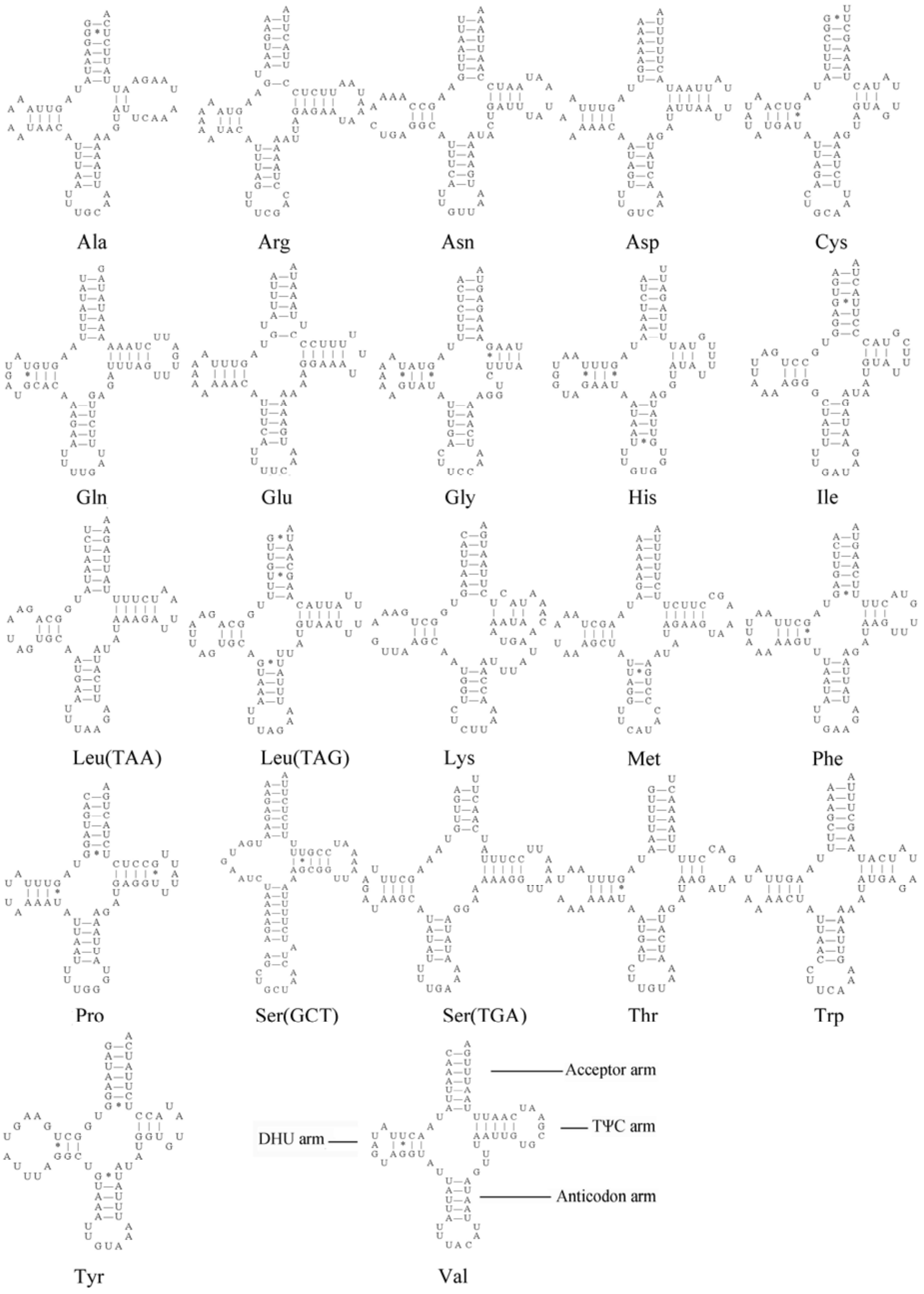

Figure 12 Putative secondary structures of mitochondrial tRNA molecules from Ochteridae

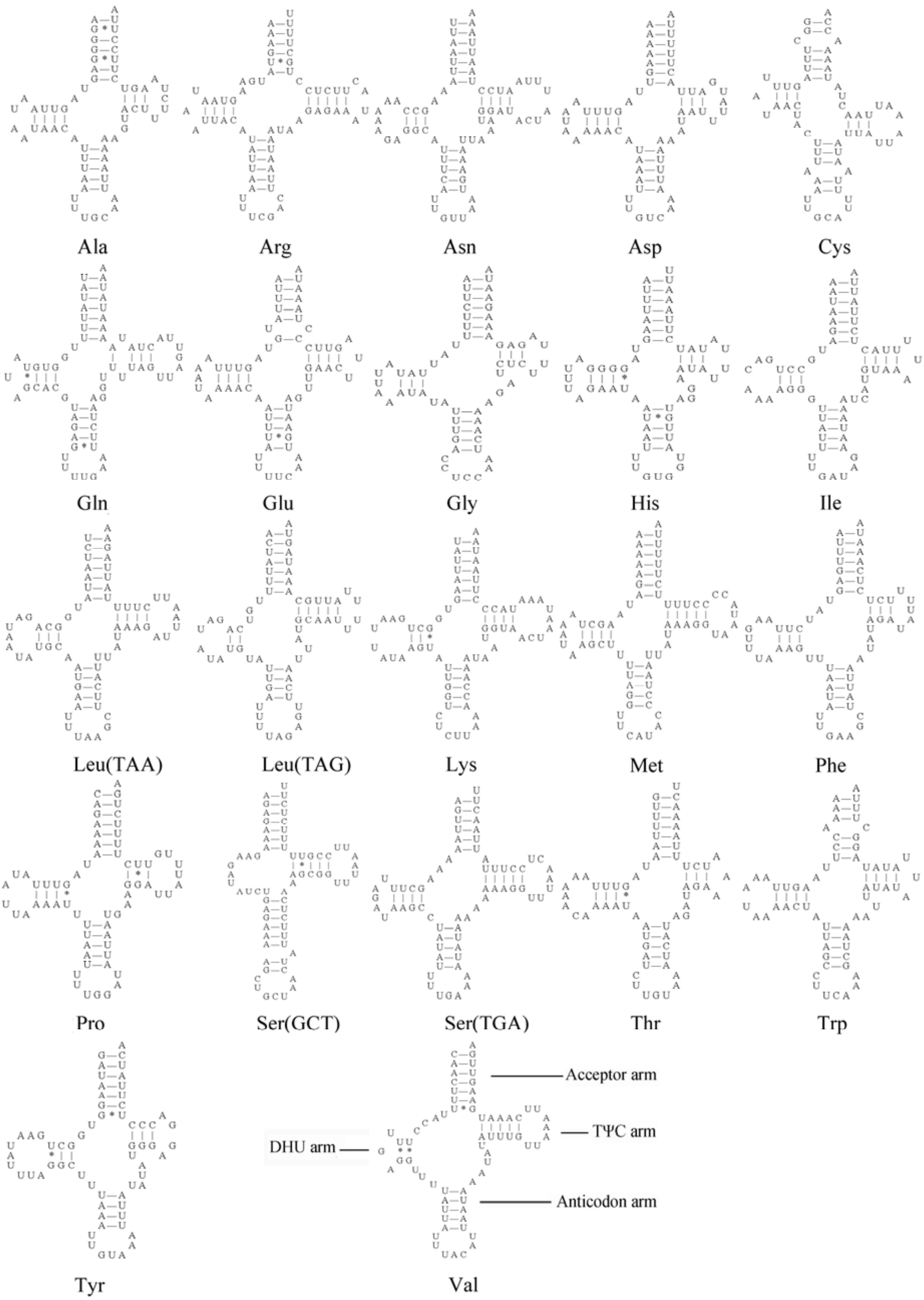

Figure 13 Putative secondary structures of mitochondrial tRNA molecules from Pleidae

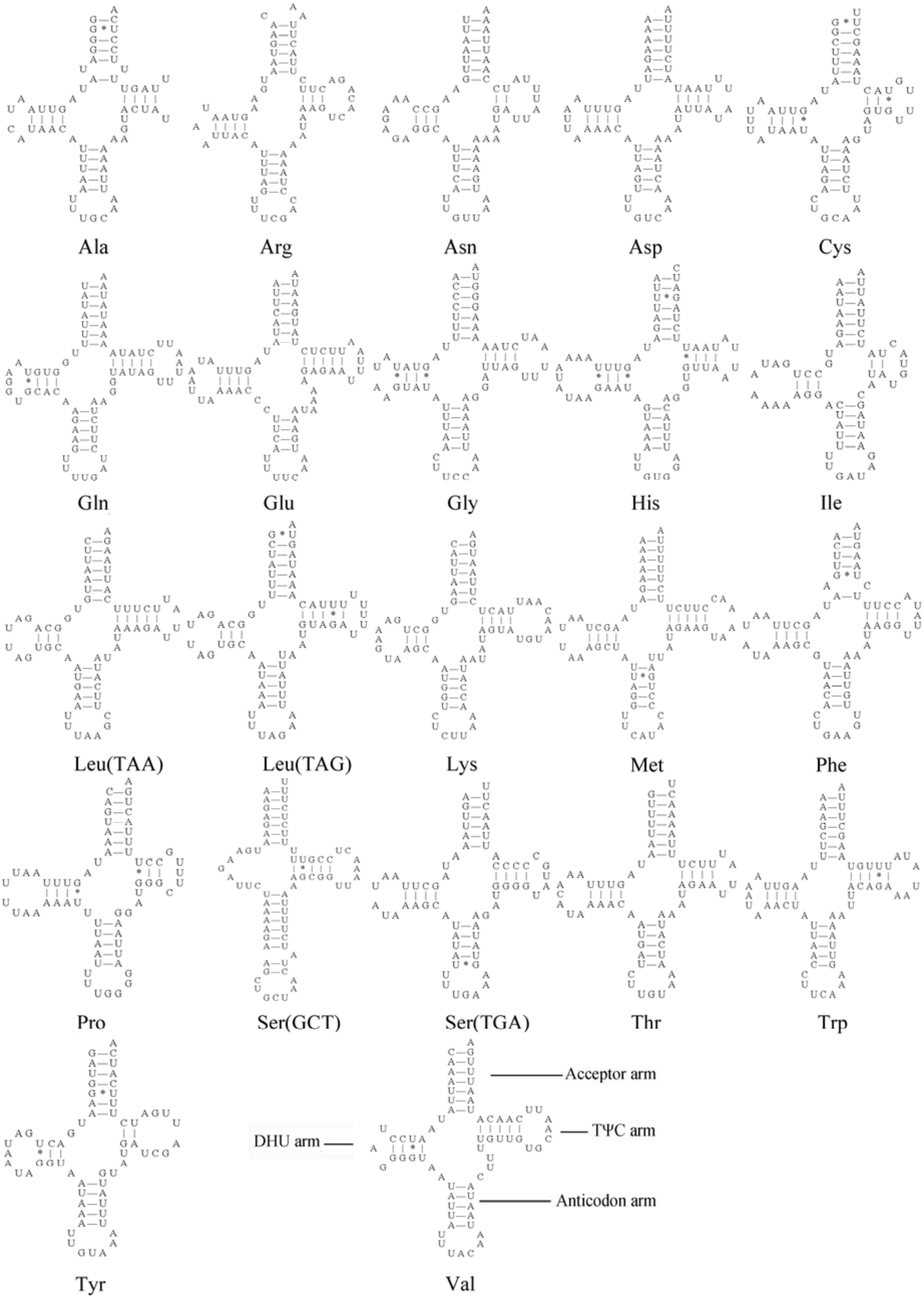

Figure 14 Putative secondary structures of mitochondrial tRNA molecules from Reduviidae

* indicated the G=U match.

Codon usage of protein coding genes

The most frequent codons do not always correspond with their cognates in the mitochondrial tRNAs. It appears that the recognition of the cognates between the anticodons and the codons is not related to translation efficiency. Furthermore, the most infrequently used fourfold degenerate codons are NNG and the most frequently used codons are NNA.

Table 1 Condon Usage of Aphelocheiridae

		N	RSCU	N+	RSCU +	N−	RSCU −			N	RSCU	N+	RSCU +	N−	RSCU −
Phe	UUU	**292**	**1.67**	**129**	**1.5**	**163**	**1.83**	Ser	UCU	91	2.01	16	0.63	**75**	**3.73**
	UUC	58	0.33	43	0.5	15	0.17		UCC	12	0.26	6	0.24	6	0.3
Leu	UUA	**344**	**4.02**	**173**	**3.77**	**171**	**4.29**		UCA	**115**	**2.53**	**96**	**3.8**	19	0.94
	UUG	43	0.5	11	0.24	32	0.8		UCG	2	0.04	2	0.08	0	0
	CUU	39	0.46	16	0.35	**23**	**0.58**	Pro	CCU	47	1.45	28	1.17	**19**	**2.24**
	CUC	3	0.04	2	0.04	1	0.03		CCC	10	0.31	7	0.29	3	0.35
	CUA	**74**	**0.86**	**63**	**1.37**	11	0.28		CCA	**72**	**2.22**	**61**	**2.54**	11	1.29
	CUG	11	0.13	10	0.22	1	0.03		CCG	1	0.03	0	0	1	0.12
Ile	AUU	**313**	**1.74**	**208**	**1.68**	**105**	**1.88**	Thr	ACU	54	1.14	30	0.8	**24**	**2.4**
	AUC	46	0.26	39	0.32	7	0.12		ACC	8	0.17	7	0.19	1	0.1
Met	AUA	**260**	**1.66**	**191**	**1.8**	**69**	**1.37**		ACA	**124**	**2.61**	**111**	**2.96**	13	1.3
	AUG	53	0.34	21	0.2	32	0.63		ACG	4	0.08	2	0.05	2	0.2
Val	GUU	**90**	**1.9**	26	1.03	**64**	**2.91**	Ala	GCU	**65**	**1.76**	27	1.08	**38**	**3.17**
	GUC	6	0.13	4	0.16	2	0.09		GCC	17	0.46	13	0.52	4	0.33
	GUA	82	1.74	**68**	**2.69**	14	0.64		GCA	64	1.73	**59**	**2.36**	5	0.42
	GUG	11	0.23	3	0.12	8	0.36		GCG	2	0.05	1	0.04	1	0.08
Tyr	UAU	**125**	**1.6**	**57**	**1.54**	**68**	**1.66**	Cys	UGU	**43**	**1.69**	**11**	**1.38**	**32**	**1.83**
	UAC	31	0.4	17	0.46	14	0.34		UGC	8	0.31	5	0.62	3	0.17
TER	UAA	6	1.71	3	1.5	3	2	Trp	UGA	**84**	**1.65**	**66**	**1.86**	**18**	**1.16**
	UAG	1	0.29	1	0.5	0	0		UGG	18	0.35	5	0.14	13	0.84
His	CAU	**52**	**1.46**	**38**	**1.33**	**14**	**2**	Arg	CGU	17	1.26	4	0.46	**13**	**2.74**
	CAC	19	0.54	19	0.67	0	0		CGC	0	0	0	0	0	0
Gln	CAA	**51**	**1.76**	**42**	**1.95**	**9**	**1.2**		CGA	**32**	**2.37**	**29**	**3.31**	3	0.63
	CAG	7	0.24	1	0.05	6	0.8		CGG	5	0.37	2	0.23	3	0.63
Asn	AAU	**142**	**1.52**	**88**	**1.43**	**54**	**1.69**	Ser	AGU	47	1.04	15	0.59	**32**	**1.59**
	AAC	45	0.48	35	0.57	10	0.31		AGC	6	0.13	3	0.12	3	0.15
Lys	AAA	**82**	**1.59**	**58**	**1.68**	**24**	**1.41**		AGA	**90**	**1.98**	**64**	**2.53**	26	1.29
	AAG	21	0.41	11	0.32	10	0.59		AGG	0	0	0	0	0	0
Asp	GAU	**49**	**1.42**	**28**	**1.27**	**21**	**1.68**	Gly	GGU	64	1.29	21	0.69	**43**	**2.23**
	GAC	20	0.58	16	0.73	4	0.32		GGC	15	0.3	5	0.16	10	0.52
Glu	GAA	**69**	**1.7**	**53**	**1.89**	**16**	**1.28**		GGA	**86**	**1.73**	**78**	**2.56**	8	0.42
	GAG	12	0.3	3	0.11	9	0.72		GGG	34	0.68	18	0.59	16	0.83

Table 2 Condon Usage of Belostomatidae

		N	RSCU	N+	RSCU +	N−	RSCU −			N	RSCU	N+	RSCU +	N−	RSCU −
Phe	UUU	**265**	**1.52**	**93**	**1.13**	**265**	**1.52**	Ser	UCU	**106**	**2.36**	20	0.83	**106**	**2.36**
	UUC	83	0.48	72	0.87	83	0.48		UCC	17	0.38	11	0.46	17	0.38
Leu	UUA	**239**	**2.71**	**116**	**2.38**	**239**	**2.71**		UCA	94	2.09	**83**	**3.44**	94	2.09
	UUG	81	0.92	16	0.33	81	0.92		UCG	7	0.16	4	0.17	7	0.16
	CUU	64	0.73	30	0.62	64	0.73	Pro	CCU	36	1.09	11	0.45	36	1.09
	CUC	19	0.22	17	0.35	19	0.22		CCC	31	0.94	27	1.1	31	0.94
	CUA	**104**	**1.18**	**95**	**1.95**	**104**	**1.18**		CCA	**52**	**1.58**	**48**	**1.96**	**52**	**1.58**
	CUG	22	0.25	18	0.37	22	0.25		CCG	13	0.39	12	0.49	13	0.39
Ile	AUU	**259**	**1.63**	**162**	**1.53**	**259**	**1.63**	Thr	ACU	80	1.45	48	1.06	80	1.45
	AUC	59	0.37	50	0.47	59	0.37		ACC	37	0.67	35	0.77	37	0.67
Met	AUA	**205**	**1.54**	**152**	**1.64**	**205**	**1.54**		ACA	**97**	**1.76**	**91**	**2.01**	**97**	**1.76**
	AUG	61	0.46	33	0.36	61	0.46		ACG	7	0.13	7	0.15	7	0.13
Val	GUU	**94**	**1.72**	34	1.05	**94**	**1.72**	Ala	GCU	67	1.48	29	0.93	67	1.48
	GUC	14	0.26	10	0.31	14	0.26		GCC	39	0.86	30	0.96	39	0.86
	GUA	92	1.69	**74**	**2.29**	92	1.69		GCA	67	1.48	**61**	**1.95**	67	1.48
	GUG	18	0.33	11	0.34	18	0.33		GCG	8	0.18	5	0.16	8	0.18
Tyr	UAU	**108**	**1.4**	**39**	**1.08**	**108**	**1.4**	Cys	UGU	**46**	**1.7**	**10**	**1.43**	**46**	**1.7**
	UAC	46	0.6	33	0.92	46	0.6		UGC	8	0.3	4	0.57	8	0.3
TER	UAA	6	1.5	4	1.6	6	1.5	Trp	UGA	**80**	**1.57**	**59**	**1.66**	**80**	**1.57**
	UAG	2	0.5	1	0.4	2	0.5		UGG	22	0.43	12	0.34	22	0.43
His	CAU	**43**	**1.12**	29	0.95	**43**	**1.12**	Arg	CGU	16	1.16	6	0.69	16	1.16
	CAC	34	0.88	**32**	**1.05**	34	0.88		CGC	5	0.36	4	0.46	5	0.36
Gln	CAA	**50**	**1.64**	**39**	**1.77**	**50**	**1.64**		CGA	**23**	**1.67**	**22**	**2.51**	**23**	**1.67**
	CAG	11	0.36	5	0.23	11	0.36		CGG	11	0.8	3	0.34	11	0.8
Asn	AAU	**102**	**1.42**	**61**	**1.26**	**102**	**1.42**	Ser	AGU	45	1	14	0.58	45	1
	AAC	42	0.58	36	0.74	42	0.58		AGC	19	0.42	13	0.54	19	0.42
Lys	AAA	**57**	**1.19**	**52**	**1.55**	**57**	**1.19**		AGA	**69**	**1.54**	**48**	**1.99**	**69**	**1.54**
	AAG	39	0.81	15	0.45	39	0.81		AGG	2	0.04	0	0	2	0.04
Asp	GAU	**46**	**1.28**	**24**	**1.04**	**46**	**1.28**	Gly	GGU	59	1.04	11	0.33	59	1.04
	GAC	26	0.72	22	0.96	26	0.72		GGC	20	0.35	8	0.24	20	0.35
Glu	GAA	**63**	**1.58**	**47**	**1.81**	**63**	**1.58**		GGA	**93**	**1.65**	**84**	**2.55**	**93**	**1.65**
	GAG	17	0.43	5	0.19	17	0.43		GGG	54	0.96	29	0.88	54	0.96

Table 3 Condon Usage of Corixidae

		N	RSCU	N+	RSCU +	N−	RSCU −			N	RSCU	N+	RSCU +	N−	RSCU −
Phe	UUU	**262**	**1.65**	**112**	**1.39**	**150**	**1.92**	Ser	UCU	89	2.11	21	0.86	**68**	**3.86**
	UUC	55	0.35	49	0.61	6	0.08		UCC	10	0.24	5	0.2	5	0.28
Leu	UUA	**338**	**4.1**	**186**	**4.04**	**152**	**4.16**		UCA	**111**	**2.64**	**86**	**3.51**	25	1.42
	UUG	50	0.61	13	0.28	37	1.01		UCG	9	0.21	5	0.2	4	0.23
	CUU	48	0.58	30	0.65	**18**	**0.49**	Pro	CCU	52	1.61	31	1.29	**21**	**2.55**
	CUC	1	0.01	0	0	1	0.03		CCC	18	0.56	14	0.58	4	0.48
	CUA	**54**	**0.65**	**44**	**0.96**	10	0.27		CCA	**54**	**1.67**	**48**	**2**	6	0.73
	CUG	4	0.05	3	0.07	1	0.03		CCG	5	0.16	3	0.12	2	0.24
Ile	AUU	**375**	**1.84**	**237**	**1.8**	**138**	**1.92**	Thr	ACU	65	1.49	41	1.21	**24**	**2.4**
	AUC	32	0.16	26	0.2	6	0.08		ACC	14	0.32	8	0.24	6	0.6
Met	AUA	**297**	**1.81**	**195**	**1.87**	**102**	**1.7**		ACA	**92**	**2.1**	**84**	**2.49**	8	0.8
	AUG	32	0.19	14	0.13	18	0.3		ACG	4	0.09	2	0.06	2	0.2
Val	GUU	66	1.48	25	0.98	**41**	**2.16**	Ala	GCU	53	1.45	20	0.79	**33**	**2.93**
	GUC	14	0.31	6	0.24	8	0.42		GCC	22	0.6	19	0.75	3	0.27
	GUA	**81**	**1.82**	**65**	**2.55**	16	0.84		GCA	**70**	**1.92**	**61**	**2.42**	9	0.8
	GUG	17	0.38	6	0.24	11	0.58		GCG	1	0.03	1	0.04	0	0
Tyr	UAU	**148**	**1.8**	**63**	**1.64**	**85**	**1.95**	Cys	UGU	**35**	**1.63**	**8**	**1.33**	**27**	**1.74**
	UAC	16	0.2	14	0.36	2	0.05		UGC	8	0.37	4	0.67	4	0.26
TER	UAA	5	1.25	4	1.33	1	1	Trp	UGA	**86**	**1.72**	**63**	**1.8**	23	1.53
	UAG	3	0.75	2	0.67	1	1		UGG	14	0.28	7	0.2	7	0.47
His	CAU	**38**	**1.06**	27	0.96	**11**	**1.38**	Arg	CGU	13	0.96	5	0.57	**8**	**1.68**
	CAC	34	0.94	**29**	**1.04**	5	0.62		CGC	3	0.22	1	0.11	2	0.42
Gln	CAA	**55**	**1.83**	**43**	**1.95**	**12**	**1.5**		CGA	**31**	**2.3**	**27**	**3.09**	4	0.84
	CAG	5	0.17	1	0.05	4	0.5		CGG	7	0.52	2	0.23	5	1.05
Asn	AAU	**185**	**1.78**	**113**	**1.69**	**72**	**1.95**	Ser	AGU	21	0.5	8	0.33	13	0.74
	AAC	23	0.22	21	0.31	2	0.05		AGC	2	0.05	1	0.04	1	0.06
Lys	AAA	**90**	**1.73**	**65**	**1.91**	**25**	**1.39**		AGA	**95**	**2.26**	**70**	**2.86**	**25**	**1.42**
	AAG	14	0.27	3	0.09	11	0.61		AGG	0	0	0	0	0	0
Asp	GAU	**45**	**1.41**	**26**	**1.27**	**19**	**1.65**	Gly	GGU	68	1.28	29	0.93	**39**	**1.79**
	GAC	19	0.59	15	0.73	4	0.35		GGC	13	0.25	3	0.1	10	0.46
Glu	GAA	**66**	**1.67**	**48**	**1.88**	**18**	**1.29**		GGA	**107**	**2.02**	**83**	**2.66**	24	1.1
	GAG	13	0.33	3	0.12	10	0.71		GGG	24	0.45	10	0.32	14	0.64

Table 4 Condon Usage of Fulgoridae

		N	RSCU	N+	RSCU +	N−	RSCU −			N	RSCU	N+	RSCU +	N−	RSCU −
Phe	UUU	413	1.74	95	1.28	318	1.94	Ser	UCU	96	2.03	25	0.88	71	3.71
	UUC	63	0.26	54	0.72	9	0.06		UCC	12	0.25	8	0.28	4	0.21
Leu	UUA	228	3.29	108	3.13	120	3.44		UCA	148	3.12	137	4.85	11	0.58
	UUG	54	0.78	3	0.09	51	1.46		UCG	3	0.06	0	0	3	0.16
	CUU							Pro	CCU						
	CUC	52	0.75	18	0.52	34	0.98		CCC	36	1.09	13	0.5	23	3.41
	CUA	7	0.1	7	0.2	0	0		CCA	18	0.55	15	0.57	3	0.44
	CUG	69	1	68	1.97	1	0.03		CCG	77	2.33	77	2.93	0	0
Ile	AUU	6	0.09	3	0.09	3	0.09	Thr	ACU	1	0.03	0	0	1	0.15
	AUC								ACC						
Met	AUA	277	1.53	173	1.35	104	1.96		ACA	53	1.06	33	0.74	20	3.64
	AUG	85	0.47	83	0.65	2	0.04		ACG	24	0.48	22	0.49	2	0.36
Val	GUU	309	1.82	275	1.89	34	1.39	Ala	GCU	121	2.42	121	2.72	0	0
	GUC	31	0.18	16	0.11	15	0.61		GCC	2	0.04	2	0.04	0	0
	GUA								GCA						
	GUG	79	1.95	11	0.57	68	3.2		GCG	22	0.95	7	0.39	15	2.73
Tyr	UAU	117	1.61	29	1.12	88	1.89	Cys	UGU	51	1.7	4	0.8	47	1.88
	UAC	28	0.39	23	0.88	5	0.11		UGC	9	0.3	6	1.2	3	0.12
TER	UAA	9	2	7	2	2	2	Trp	UGA	76	1.85	61	1.97	15	1.5
	UAG	0	0	0	0	0	0		UGG	6	0.15	1	0.03	5	0.5
His	CAU							Arg	CGU						
	CAC	34	1.05	21	0.82	13	1.86		CGC	19	1.58	2	0.3	17	3.24
Gln	CAA	31	0.95	30	1.18	1	0.14		CGA	0	0	0	0	0	0
	CAG	48	1.75	42	1.95	6	1		CGG	27	2.25	24	3.56	3	0.57
Asn	AAU	7	0.25	1	0.05	6	1	Ser	AGU	2	0.17	1	0.15	1	0.19
	AAC								AGC						
Lys	AAA	116	1.54	75	1.36	41	2		AGA	24	0.51	2	0.07	22	1.15
	AAG	35	0.46	35	0.64	0	0		AGG	1	0.02	0	0	1	0.05
Asp	GAU	135	1.79	130	1.95	5	0.56	Gly	GGU	82	1.73	54	1.91	28	1.46
	GAC	16	0.21	3	0.05	13	1.44		GGC	13	0.27	0	0	13	0.68
Glu	GAA								GGA						
	GAG	46	1.37	12	0.77	34	1.89		GGG	66	1.49	12	0.48	54	2.77

Table 5 Condon Usage of Gelastocoridae

		N	RSCU	N+	RSCU +	N−	RSCU −			N	RSCU	N+	RSCU +	N−	RSCU −
Phe	UUU	**316**	**1.67**	**115**	**1.36**	**201**	**1.91**	Ser	UCU	86	2.04	16	0.67	**70**	**3.78**
	UUC	63	0.33	54	0.64	9	0.09		UCC	16	0.38	15	0.63	1	0.05
Leu	UUA	**325**	**4**	**158**	**3.82**	**167**	**4.18**		UCA	**113**	**2.67**	**92**	**3.87**	21	1.14
	UUG	58	0.71	8	0.19	50	1.25		UCG	3	0.07	1	0.04	2	0.11
	CUU	31	0.38	16	0.39	**15**	**0.38**	Pro	CCU	55	1.62	30	1.2	**25**	**2.78**
	CUC	9	0.11	8	0.19	1	0.03		CCC	16	0.47	13	0.52	3	0.33
	CUA	**56**	**0.69**	**53**	**1.28**	3	0.08		CCA	**59**	**1.74**	**52**	**2.08**	7	0.78
	CUG	9	0.11	5	0.12	4	0.1		CCG	6	0.18	5	0.2	1	0.11
Ile	AUU	**342**	**1.77**	**227**	**1.71**	**115**	**1.92**	Thr	ACU	51	1.09	24	0.64	**27**	**2.92**
	AUC	44	0.23	39	0.29	5	0.08		ACC	15	0.32	10	0.26	5	0.54
Met	AUA	**310**	**1.8**	**231**	**1.89**	**79**	**1.58**		ACA	**118**	**2.51**	**114**	**3.02**	4	0.43
	AUG	35	0.2	14	0.11	21	0.42		ACG	4	0.09	3	0.08	1	0.11
Val	GUU	**83**	**2**	26	1.17	**57**	**2.96**	Ala	GCU	47	1.38	19	0.78	**28**	**2.87**
	GUC	5	0.12	3	0.13	2	0.1		GCC	9	0.26	7	0.29	2	0.21
	GUA	69	1.66	**55**	**2.47**	14	0.73		GCA	**74**	**2.18**	**67**	**2.76**	7	0.72
	GUG	9	0.22	5	0.22	4	0.21		GCG	6	0.18	4	0.16	2	0.21
Tyr	UAU	**123**	**1.7**	**49**	**1.42**	**74**	**1.95**	Cys	UGU	**54**	**1.77**	**11**	**1.47**	**43**	**1.87**
	UAC	22	0.3	20	0.58	2	0.05		UGC	7	0.23	4	0.53	3	0.13
TER	UAA	8	1.6	5	1.67	3	1.5	Trp	UGA	**85**	**1.7**	**69**	**1.92**	**16**	**1.14**
	UAG	2	0.4	1	0.33	1	0.5		UGG	15	0.3	3	0.08	12	0.86
His	CAU	**48**	**1.35**	**37**	**1.3**	**11**	**1.57**	Arg	CGU	10	0.73	2	0.23	**8**	**1.6**
	CAC	23	0.65	20	0.7	3	0.43		CGC	4	0.29	3	0.34	1	0.2
Gln	CAA	**54**	**1.64**	**44**	**1.87**	**10**	**1.05**		CGA	**32**	**2.33**	**27**	**3.09**	5	1
	CAG	12	0.36	3	0.13	9	0.95		CGG	9	0.65	3	0.34	6	1.2
Asn	AAU	**141**	**1.61**	**92**	**1.47**	**49**	**1.96**	Ser	AGU	26	0.62	6	0.25	20	1.08
	AAC	34	0.39	33	0.53	1	0.04		AGC	3	0.07	1	0.04	2	0.11
Lys	AAA	**86**	**1.61**	**72**	**1.92**	14	0.88		AGA	**86**	**2.04**	**59**	**2.48**	**27**	**1.46**
	AAG	21	0.39	3	0.08	**18**	**1.12**		AGG	5	0.12	0	0	5	0.27
Asp	GAU	**55**	**1.59**	**30**	**1.4**	**25**	**1.92**	Gly	GGU	62	1.22	18	0.59	**44**	**2.15**
	GAC	14	0.41	13	0.6	1	0.08		GGC	9	0.18	6	0.2	3	0.15
Glu	GAA	**64**	**1.54**	**51**	**1.82**	13	0.96		GGA	**84**	**1.65**	**69**	**2.26**	15	0.73
	GAG	19	0.46	5	0.18	**14**	**1.04**		GGG	49	0.96	29	0.95	20	0.98

Table 6 Condon Usage of Gerridae

		N	RSCU	N+	RSCU +	N−	RSCU −			N	RSCU	N+	RSCU +	N−	RSCU −
Phe	UUU	**284**	**1.65**	**105**	**1.35**	**179**	**1.88**	Ser	UCU	90	2.16	19	0.88	**71**	**3.62**
	UUC	61	0.35	50	0.65	11	0.12		UCC	15	0.36	8	0.37	7	0.36
Leu	UUA	**286**	**3.67**	**146**	**3.81**	**140**	**3.53**		UCA	**120**	**2.88**	**89**	**4.11**	31	1.58
	UUG	58	0.74	8	0.21	50	1.26		UCG	3	0.07	2	0.09	1	0.05
	CUU	**62**	**0.79**	28	0.73	**34**	**0.86**	Pro	CCU	50	1.63	31	1.33	**19**	**2.53**
	CUC	3	0.04	1	0.03	2	0.05		CCC	13	0.42	9	0.39	4	0.53
	CUA	55	0.71	**46**	**1.2**	9	0.23		CCA	**58**	**1.89**	**51**	**2.19**	7	0.93
	CUG	4	0.05	1	0.03	3	0.08		CCG	2	0.07	2	0.09	0	0
Ile	AUU	**376**	**1.82**	**251**	**1.44**	**125**	**1.95**	Thr	ACU	74	1.5	36	0.95	**38**	**3.38**
	AUC	37	0.18	34	0.2	3	0.05		ACC	9	0.18	8	0.21	1	0.09
Met	AUA	**315**	**1.82**	**238**	**1.37**	**77**	**1.6**		ACA	**112**	**2.27**	**106**	**2.79**	6	0.53
	AUG	31	0.18	12	1	19	0.4		ACG	2	0.04	2	0.05	0	0
Val	GUU	**79**	**2.08**	20	0.99	**59**	**3.32**	Ala	GCU	34	1.11	17	0.75	**17**	**2.12**
	GUC	4	0.11	2	0.1	2	0.11		GCC	26	0.85	17	0.75	9	1.12
	GUA	66	1.74	**57**	**2.81**	9	0.51		GCA	**58**	**1.89**	**54**	**2.37**	4	0.5
	GUG	3	0.08	2	0.1	1	0.06		GCG	5	0.16	3	0.13	2	0.25
Tyr	UAU	**137**	**1.69**	**50**	**1.41**	**87**	**1.91**	Cys	UGU	**41**	**1.78**	**13**	**1.62**	**28**	**1.87**
	UAC	25	0.31	21	0.59	4	0.09		UGC	5	0.22	3	0.38	2	0.13
TER	UAA	4	1.6	2	0.09	2	1.33	Trp	UGA	**87**	**1.83**	**67**	**2.91**	**20**	**1.54**
	UAG	1	0.4	0	0	1	0.67		UGG	8	0.17	2	1	6	0.46
His	CAU	**42**	**1.24**	**30**	**1.13**	**12**	**1.6**	Arg	CGU	19	1.49	5	0.38	**14**	**2.95**
	CAC	26	0.76	23	0.87	3	0.4		CGC	0	0	0	0	0	0
Gln	CAA	**58**	**1.84**	**48**	**2**	**10**	**1.33**		CGA	**30**	**2.35**	**25**	**1.92**	5	1.05
	CAG	5	0.16	0	0	5	0.67		CGG	2	0.16	2	0.15	0	0
Asn	AAU	**169**	**1.58**	**104**	**1.43**	**65**	**1.88**	Ser	AGU	25	0.6	10	0.46	15	0.76
	AAC	45	0.42	41	0.57	4	0.12		AGC	7	0.17	2	0.09	5	0.25
Lys	AAA	**112**	**1.79**	**91**	**1.94**	**21**	**1.35**		AGA	**73**	**1.75**	**46**	**3.54**	**27**	**1.38**
	AAG	13	0.21	3	0.06	10	0.65		AGG	0	0	0	0	0	0
Asp	GAU	**46**	**1.51**	**24**	**1.26**	**22**	**1.91**	Gly	GGU	80	1.56	25	0.81	**55**	**2.68**
	GAC	15	0.49	14	0.74	1	0.09		GGC	3	0.06	0	0	3	0.15
Glu	GAA	**71**	**1.61**	**55**	**1.83**	**16**	**1.14**		GGA	**94**	**1.83**	**83**	**2.7**	11	0.54
	GAG	17	0.39	5	0.17	12	0.86		GGG	28	0.55	15	0.49	13	0.63

Table 7 Condon Usage of Hydrometridae

		N	RSCU	N+	RSCU +	N−	RSCU −			N	RSCU	N+	RSCU +	N−	RSCU −
Phe	UUU	**274**	**1.77**	**115**	**1.58**	**159**	**1.94**	Ser	UCU	88	2.14	23	1.01	**65**	**3.56**
	UUC	36	0.23	31	0.42	5	0.06		UCC	8	0.19	7	0.31	1	0.05
Leu	UUA	**333**	**4.32**	**158**	**4.27**	**175**	**4.38**		UCA	**129**	**3.14**	**106**	**4.63**	23	1.26
	UUG	32	0.42	4	0.11	28	0.7		UCG	2	0.05	1	0.04	1	0.05
	CUU	35	0.45	13	0.35	**22**	**0.55**	Pro	CCU	40	1.34	22	0.99	**18**	**2.4**
	CUC	3	0.04	1	0.03	2	0.05		CCC	11	0.37	7	0.31	4	0.53
	CUA	**57**	**0.74**	**45**	**1.22**	12	0.3		CCA	**61**	**2.05**	**53**	**2.38**	8	1.07
	CUG	2	0.03	1	0.03	1	0.03		CCG	7	0.24	7	0.31	0	0
Ile	AUU	**437**	**1.85**	**291**	**1.8**	**146**	**1.96**	Thr	ACU	58	1.46	26	0.87	**32**	**3.28**
	AUC	36	0.15	33	0.2	3	0.04		ACC	18	0.45	14	0.47	4	0.41
Met	AUA	**363**	**1.85**	**261**	**1.91**	**102**	**1.73**		ACA	**82**	**2.06**	**79**	**2.63**	3	0.31
	AUG	29	0.15	13	0.09	16	0.27		ACG	1	0.03	1	0.03	0	0
Val	GUU	71	1.82	30	1.3	**41**	**2.56**	Ala	GCU	42	1.7	24	1.32	**18**	**2.77**
	GUC	5	0.13	3	0.13	2	0.12		GCC	8	0.32	6	0.33	2	0.31
	GUA	**75**	**1.92**	**57**	**2.48**	18	1.12		GCA	**47**	**1.9**	**43**	**2.36**	4	0.62
	GUG	5	0.13	2	0.09	3	0.19		GCG	2	0.08	0	0	2	0.31
Tyr	UAU	**160**	**1.82**	**68**	**1.68**	**92**	**1.94**	Cys	UGU	**33**	**1.83**	**8**	**1.6**	**25**	**1.92**
	UAC	16	0.18	13	0.32	3	0.06		UGC	3	0.17	2	0.4	1	0.08
TER	UAA	4	1.33	2	1	2	2	Trp	UGA	**80**	**1.82**	**62**	**1.88**	**18**	**1.64**
	UAG	2	0.67	2	1	0	0		UGG	8	0.18	4	0.12	4	0.36
His	CAU	**37**	**1.17**	23	1	**14**	**1.65**	Arg	CGU	19	1.52	2	0.26	**17**	**3.58**
	CAC	26	0.83	23	1	3	0.35		CGC	2	0.16	2	0.26	0	0
Gln	CAA	**51**	**1.79**	**40**	**1.86**	**11**	**1.57**		CGA	**27**	**2.16**	**27**	**3.48**	0	0
	CAG	6	0.21	3	0.14	3	0.43		CGG	2	0.16	0	0	2	0.42
Asn	AAU	**184**	**1.53**	**108**	**1.38**	**76**	**1.81**	Ser	AGU	16	0.39	2	0.09	14	0.77
	AAC	56	0.47	48	0.62	8	0.19		AGC	0	0	0	0	0	0
Lys	AAA	**117**	**1.68**	**89**	**1.82**	**28**	**1.37**		AGA	**83**	**2.02**	**44**	**1.92**	**39**	**2.14**
	AAG	22	0.32	9	0.18	13	0.63		AGG	3	0.07	0	0	3	0.16
Asp	GAU	**41**	**1.44**	**25**	**1.32**	**16**	**1.68**	Gly	GGU	70	1.56	19	0.68	**51**	**3.04**
	GAC	16	0.56	13	0.68	3	0.32		GGC	5	0.11	0	0	5	0.3
Glu	GAA	**70**	**1.82**	**46**	**1.88**	**24**	**1.71**		GGA	**84**	**1.88**	**76**	**2.71**	8	0.48
	GAG	7	0.18	3	0.12	4	0.29		GGG	20	0.45	17	0.61	3	0.18

Table 8 Condon Usage of Leptopodidae

		N	RSCU	N+	RSCU +	N−	RSCU −			N	RSCU	N+	RSCU +	N−	RSCU −
Phe	UUU	**249**	**1.49**	73	1	**176**	**1.87**	Ser	UCU	98	2.28	15	0.65	**83**	**4.2**
	UUC	85	0.51	73	1	12	0.13		UCC	16	0.37	11	0.47	5	0.25
Leu	UUA	**256**	**3.15**	**122**	**3.08**	**134**	**3.22**		UCA	**105**	**2.44**	**89**	**3.83**	16	0.81
	UUG	75	0.92	4	0.1	71	1.7		UCG	3	0.07	0	0	3	0.15
	CUU	46	0.57	10	0.25	**36**	**0.86**	Pro	CCU	50	1.53	26	1.04	**24**	**3.1**
	CUC	9	0.11	4	0.1	5	0.12		CCC	13	0.4	11	0.44	2	0.26
	CUA	**95**	**1.17**	**92**	**2.32**	3	0.07		CCA	**62**	**1.89**	**58**	**2.32**	4	0.52
	CUG	7	0.09	6	0.15	1	0.02		CCG	6	0.18	5	0.2	1	0.13
Ile	AUU	**250**	**1.57**	**149**	**1.39**	**101**	**1.94**	Thr	ACU	59	1.03	33	0.68	**26**	**2.97**
	AUC	68	0.43	65	0.61	3	0.06		ACC	20	0.35	18	0.37	2	0.23
Met	AUA	**289**	**1.8**	**236**	**1.86**	**53**	**1.58**		ACA	**142**	**2.48**	**136**	**2.8**	6	0.69
	AUG	32	0.2	18	0.14	14	0.42		ACG	8	0.14	7	0.14	1	0.11
Val	GUU	100	1.75	20	0.67	**80**	**2.96**	Ala	GCU	**59**	**1.74**	23	0.98	**36**	**3.43**
	GUC	6	0.11	2	0.07	4	0.15		GCC	19	0.56	17	0.72	2	0.19
	GUA	**111**	**1.95**	**94**	**3.13**	17	0.63		GCA	56	1.65	**53**	**2.26**	3	0.29
	GUG	11	0.19	4	0.13	7	0.26		GCG	2	0.06	1	0.04	1	0.1
Tyr	UAU	**118**	**1.53**	**33**	**1.03**	**85**	**1.89**	Cys	UGU	**47**	**1.74**	**10**	**1.33**	**37**	**1.9**
	UAC	36	0.47	31	0.97	5	0.11		UGC	7	0.26	5	0.67	2	0.1
TER	UAA	7	1.4	5	1.67	2	1	Trp	UGA	**77**	**1.57**	**67**	**1.91**	10	0.71
	UAG	3	0.6	1	0.33	2	1		UGG	21	0.43	3	0.09	**18**	**1.29**
His	CAU	**38**	**1.07**	23	0.82	**15**	**2**	Arg	CGU	16	1.16	2	0.24	**14**	**2.67**
	CAC	33	0.93	**33**	**1.18**	0	0		CGC	1	0.07	0	0	1	0.19
Gln	CAA	**52**	**1.7**	**45**	**1.91**	7	1		CGA	**34**	**2.47**	**30**	**3.53**	4	0.76
	CAG	9	0.3	2	0.09	7	1		CGG	4	0.29	2	0.24	2	0.38
Asn	AAU	**117**	**1.3**	61	0.99	**56**	**1.96**	Ser	AGU	34	0.79	2	0.09	**32**	**1.62**
	AAC	63	0.7	**62**	**1.01**	1	0.04		AGC	2	0.05	1	0.04	1	0.05
Lys	AAA	**83**	**1.5**	**74**	**1.8**	9	0.62		AGA	**85**	**1.98**	**68**	**2.92**	17	0.86
	AAG	28	0.5	8	0.2	**20**	**1.38**		AGG	1	0.02	0	0	1	0.05
Asp	GAU	**46**	**1.3**	18	0.84	**28**	**2**	Gly	GGU	81	1.58	17	0.57	**64**	**3.01**
	GAC	25	0.7	**25**	**1.16**	0	0		GGC	4	0.08	3	0.1	1	0.05
Glu	GAA	**72**	**1.66**	**59**	**1.9**	**13**	**1.04**		GGA	**103**	**2.01**	**95**	**3.17**	8	0.38
	GAG	15	0.34	3	0.1	12	0.96		GGG	17	0.33	5	0.17	12	0.56

Table 9 Condon Usage of Naucoridae

		N	RSCU	N+	RSCU +	N−	RSCU −			N	RSCU	N+	RSCU +	N−	RSCU −
Phe	UUU	**234**	**1.44**	**81**	**1.01**	**153**	**1.85**	Ser	UCU	105	2.26	18	0.71	**87**	**4.12**
	UUC	91	0.56	79	0.99	12	0.15		UCC	18	0.39	13	0.51	5	0.24
Leu	UUA	**265**	**2.99**	**124**	**2.68**	**141**	**3.33**		UCA	**106**	**2.29**	**85**	**3.37**	21	0.99
	UUG	64	0.72	19	0.41	45	1.06		UCG	3	0.06	2	0.08	1	0.05
	CUU	79	0.89	30	0.65	**49**	**1.16**	Pro	CCU	43	1.29	21	0.85	**22**	**2.59**
	CUC	13	0.15	7	0.15	6	0.14		CCC	27	0.81	21	0.85	6	0.71
	CUA	**87**	**0.98**	**77**	**1.66**	10	0.24		CCA	**59**	**1.77**	**54**	**2.18**	5	0.59
	CUG	24	0.27	21	0.45	3	0.07		CCG	4	0.12	3	0.12	1	0.12
Ile	AUU	**284**	**1.62**	**176**	**1.49**	**108**	**1.89**	Thr	ACU	65	1.38	31	0.84	**34**	**3.32**
	AUC	66	0.38	60	0.51	6	0.11		ACC	25	0.53	22	0.59	3	0.29
Met	AUA	**238**	**1.61**	**181**	**1.72**	**57**	**1.34**		ACA	**97**	**2.05**	**93**	**2.51**	4	0.39
	AUG	58	0.39	30	0.28	28	0.66		ACG	2	0.04	2	0.05	0	0
Val	GUU	67	1.29	12	0.41	**55**	**2.42**	Ala	GCU	55	1.43	18	0.67	**37**	**3.22**
	GUC	11	0.21	3	0.1	8	0.35		GCC	23	0.6	20	0.74	3	0.26
	GUA	**113**	**2.17**	**94**	**3.21**	19	0.84		GCA	**74**	**1.92**	**69**	**2.56**	5	0.43
	GUG	17	0.33	8	0.27	9	0.4		GCG	2	0.05	1	0.04	1	0.09
Tyr	UAU	**121**	**1.56**	**41**	**1.22**	**80**	**1.82**	Cys	UGU	**39**	**1.42**	4	0.5	**35**	**1.79**
	UAC	34	0.44	26	0.78	8	0.18		UGC	16	0.58	**12**	**1.5**	4	0.21
TER	UAA	7	1.56	4	1.6	3	1.5	Trp	UGA	**84**	**1.71**	**64**	**1.86**	**20**	**1.38**
	UAG	2	0.44	1	0.4	1	0.5		UGG	14	0.29	5	0.14	9	0.62
His	CAU	34	0.96	21	0.74	**13**	**1.86**	Arg	CGU	18	1.29	4	0.46	**14**	**2.67**
	CAC	**37**	**1.04**	**36**	**1.26**	1	0.14		CGC	2	0.14	1	0.11	1	0.19
Gln	CAA	**48**	**1.52**	**41**	**1.82**	7	0.78		CGA	**32**	**2.29**	**28**	**3.2**	4	0.76
	CAG	15	0.48	4	0.18	**11**	**1.22**		CGG	4	0.29	2	0.23	2	0.38
Asn	AAU	**112**	**1.43**	**62**	**1.23**	**50**	**1.79**	Ser	AGU	25	0.54	8	0.32	17	0.8
	AAC	45	0.57	39	0.77	6	0.21		AGC	8	0.17	5	0.2	3	0.14
Lys	AAA	**75**	**1.56**	**67**	**1.74**	8	0.84		AGA	**94**	**2.03**	**71**	**2.81**	**23**	**1.09**
	AAG	21	0.44	10	0.26	**11**	**1.16**		AGG	12	0.26	0	0	12	0.57
Asp	GAU	**45**	**1.25**	22	0.92	**23**	**1.92**	Gly	GGU	70	1.22	16	0.45	**54**	**2.43**
	GAC	27	0.75	**26**	**1.08**	1	0.08		GGC	12	0.21	7	0.2	5	0.22
Glu	GAA	**67**	**1.54**	**55**	**1.9**	12	0.83		GGA	**111**	**1.93**	**97**	**2.75**	14	0.63
	GAG	20	0.46	3	0.1	**17**	**1.17**		GGG	37	0.64	21	0.6	16	0.72

Table 10 Condon Usage of Nepidae

		N	RSCU	N+	RSCU +	N−	RSCU −			N	RSCU	N+	RSCU +	N−	RSCU −
Phe	UUU	**256**	**1.48**	74	0.94	**182**	**1.92**	Ser	UCU	**110**	**2.54**	17	0.74	**93**	**4.54**
	UUC	91	0.52	**83**	**1.06**	8	0.08		UCC	19	0.44	15	0.66	4	0.2
Leu	UUA	**234**	**2.86**	**129**	**3.01**	**105**	**2.69**		UCA	84	1.94	**76**	**3.32**	8	0.39
	UUG	68	0.83	6	0.14	62	1.59		UCG	5	0.12	2	0.09	3	0.15
	CUU	80	0.98	22	0.51	**58**	**1.49**	Pro	CCU	**54**	**1.66**	26	1.06	**28**	**3.5**
	CUC	10	0.12	8	0.19	2	0.05		CCC	21	0.65	20	0.82	1	0.12
	CUA	**90**	**1.1**	**88**	**2.05**	2	0.05		CCA	51	1.57	**49**	**2**	2	0.25
	CUG	9	0.11	4	0.09	5	0.13		CCG	4	0.12	3	0.12	1	0.12
Ile	AUU	**257**	**1.55**	**167**	**1.42**	**90**	**1.88**	Thr	ACU	69	1.27	34	0.76	**35**	**3.68**
	AUC	74	0.45	68	0.58	6	0.12		ACC	25	0.46	25	0.56	0	0
Met	AUA	**251**	**1.67**	**206**	**1.83**	**45**	**1.18**		ACA	**120**	**2.2**	**117**	**2.6**	3	0.32
	AUG	50	0.33	19	0.17	31	0.82		ACG	4	0.07	4	0.09	0	0
Val	GUU	86	1.61	9	0.33	**77**	**2.96**	Ala	GCU	**73**	**1.67**	32	1.02	**41**	**3.28**
	GUC	9	0.17	7	0.25	2	0.08		GCC	27	0.62	26	0.83	1	0.08
	GUA	**103**	**1.93**	**85**	**3.09**	18	0.69		GCA	69	1.58	**64**	**2.05**	5	0.4
	GUG	16	0.3	9	0.33	7	0.27		GCG	6	0.14	3	0.1	3	0.24
Tyr	UAU	**118**	**1.53**	**38**	**1.1**	**80**	**1.88**	Cys	UGU	**52**	**1.73**	**10**	**1.18**	**42**	**1.95**
	UAC	36	0.47	31	0.9	5	0.12		UGC	8	0.27	7	0.82	1	0.05
TER	UAA	5	1.43	4	2	1	0.67	Trp	UGA	**83**	**1.68**	**66**	**1.91**	**17**	**1.13**
	UAG	2	0.57	0	0	2	1.33		UGG	16	0.32	3	0.09	13	0.87
His	CAU	**38**	**1.04**	23	0.81	**15**	**1.88**	Arg	CGU	21	1.5	4	0.44	**17**	**3.4**
	CAC	35	0.96	**34**	**1.19**	1	0.12		CGC	3	0.21	3	0.33	0	0
Gln	CAA	**51**	**1.57**	**44**	**1.87**	7	0.78		CGA	**30**	**2.14**	**28**	**3.11**	2	0.4
	CAG	14	0.43	3	0.13	**11**	**1.22**		CGG	2	0.14	1	0.11	1	0.2
Asn	AAU	**113**	**1.48**	**64**	**1.25**	**49**	**1.92**	Ser	AGU	37	0.85	11	0.48	**26**	**1.27**
	AAC	40	0.52	38	0.75	2	0.08		AGC	4	0.09	1	0.04	3	0.15
Lys	AAA	**75**	**1.55**	**64**	**1.78**	11	0.88		AGA	**80**	**1.84**	**61**	**2.67**	19	0.93
	AAG	22	0.45	8	0.22	**14**	**1.12**		AGG	8	0.18	0	0	8	0.39
Asp	GAU	**47**	**1.34**	20	0.93	**27**	**2**	Gly	GGU	85	1.51	16	0.5	**69**	**2.88**
	GAC	23	0.66	**23**	**1.07**	0	0		GGC	11	0.2	8	0.25	3	0.12
Glu	GAA	**58**	**1.36**	**48**	**1.66**	10	0.74		GGA	**91**	**1.62**	**81**	**2.51**	10	0.42
	GAG	27	0.64	10	0.34	**17**	**1.26**		GGG	38	0.68	24	0.74	14	0.58

Table 11 Condon Usage of Notonectidae

		N	RSCU	N+	RSCU +	N−	RSCU −			N	RSCU	N+	RSCU +	N−	RSCU −
Phe	UUU	**305**	**1.74**	**133**	**1.52**	**172**	**1.95**	Ser	UCU	103	2.39	19	0.82	**84**	**4.2**
	UUC	46	0.26	42	0.48	4	0.05		UCC	12	0.28	8	0.35	4	0.2
Leu	UUA	**298**	**3.75**	**156**	**3.73**	**142**	**3.77**		UCA	**104**	**2.41**	**91**	**3.94**	13	0.65
	UUG	47	0.59	1	0.02	46	1.22		UCG	2	0.05	2	0.09	0	0
	CUU	43	0.54	15	0.36	**28**	**0.74**	Pro	CCU	57	1.8	31	1.31	**26**	**3.25**
	CUC	7	0.09	4	0.1	3	0.08		CCC	8	0.25	7	0.29	1	0.12
	CUA	**78**	**0.98**	**73**	**1.75**	5	0.13		CCA	**61**	**1.92**	**56**	**2.36**	5	0.62
	CUG	4	0.05	2	0.05	2	0.05		CCG	1	0.03	1	0.04	0	0
Ile	AUU	**330**	**1.75**	**204**	**1.66**	**126**	**1.91**	Thr	ACU	72	1.52	40	1.09	**32**	**3.05**
	AUC	48	0.25	42	0.34	6	0.09		ACC	7	0.15	5	0.14	2	0.19
Met	AUA	**365**	**1.87**	**272**	**1.94**	**93**	**1.69**		ACA	**108**	**2.29**	**100**	**2.72**	8	0.76
	AUG	26	0.13	9	0.06	17	0.31		ACG	2	0.04	2	0.05	0	0
Val	GUU	77	1.72	13	0.54	**64**	**3.08**	Ala	GCU	42	1.28	10	0.43	**32**	**3.28**
	GUC	3	0.07	1	0.04	2	0.1		GCC	5	0.15	4	0.17	1	0.1
	GUA	**93**	**2.08**	**79**	**3.29**	14	0.67		GCA	**83**	**2.53**	**77**	**3.35**	6	0.62
	GUG	6	0.13	3	0.12	3	0.14		GCG	1	0.03	1	0.04	0	0
Tyr	UAU	**141**	**1.68**	**53**	**1.49**	**88**	**1.81**	Cys	UGU	44	1.8	11	1.69	**33**	**1.83**
	UAC	27	0.32	18	0.51	9	0.19		UGC	5	0.2	2	0.31	3	0.17
TER	UAA	7	1.4	4	1.33	3	1.5	Trp	UGA	**84**	**1.75**	**68**	**1.97**	16	1.19
	UAG	3	0.6	2	0.67	1	0.5		UGG	12	0.25	1	0.03	11	0.81
His	CAU	**47**	**1.31**	**31**	**1.11**	**16**	**2**	Arg	CGU	19	1.52	3	0.38	**16**	**3.56**
	CAC	25	0.69	25	0.89	0	0		CGC	0	0	0	0	0	0
Gln	CAA	**61**	**1.88**	**49**	**1.96**	**12**	**1.6**		CGA	**29**	**2.32**	**29**	**3.62**	0	0
	CAG	4	0.12	1	0.04	3	0.4		CGG	2	0.16	0	0	2	0.44
Asn	AAU	**139**	**1.58**	**86**	**1.45**	**53**	**1.86**	Ser	AGU	22	0.51	6	0.26	16	0.8
	AAC	37	0.42	33	0.55	4	0.14		AGC	1	0.02	0	0	1	0.05
Lys	AAA	**89**	**1.7**	**72**	**1.89**	**17**	**1.17**		AGA	**98**	**2.27**	**59**	**2.55**	**39**	**1.95**
	AAG	16	0.3	4	0.11	12	0.83		AGG	3	0.07	0	0	3	0.15
Asp	GAU	**52**	**1.55**	**28**	**1.37**	**24**	**1.85**	Gly	GGU	76	1.53	18	0.59	**58**	**3.01**
	GAC	15	0.45	13	0.63	2	0.15		GGC	1	0.02	1	0.03	0	0
Glu	GAA	**72**	**1.76**	**54**	**1.96**	**18**	**1.33**		GGA	**106**	**2.13**	**96**	**3.15**	10	0.52
	GAG	10	0.24	1	0.04	9	0.67		GGG	16	0.32	7	0.23	9	0.47

Table 12 Condon Usage of Ochteridae

		N	RSCU	N+	RSCU +	N−	RSCU −			N	RSCU	N+	RSCU +	N−	RSCU −
Phe	UUU	**278**	**1.51**	**91**	**1.06**	**187**	**1.92**	Ser	UCU	**113**	**2.42**	26	1.03	**87**	**4.02**
	UUC	89	0.49	81	0.94	8	0.08		UCC	32	0.68	27	1.07	5	0.23
Leu	UUA	**267**	**3.1**	**135**	**3**	**132**	**3.22**		UCA	85	1.82	**75**	**2.99**	10	0.46
	UUG	87	1.01	9	0.2	78	1.9		UCG	8	0.17	3	0.12	5	0.23
	CUU	64	0.74	37	0.82	**27**	**0.66**	Pro	CCU	**61**	**1.77**	28	1.11	**33**	**3.57**
	CUC	12	0.14	11	0.24	1	0.02		CCC	22	0.64	20	0.79	2	0.22
	CUA	**79**	**0.92**	**74**	**1.64**	5	0.12		CCA	48	1.39	**46**	**1.82**	2	0.22
	CUG	7	0.08	4	0.09	3	0.07		CCG	7	0.2	7	0.28	0	0
Ile	AUU	**294**	**1.59**	**190**	**1.44**	**104**	**1.96**	Thr	ACU	55	1.1	31	0.74	**24**	**3**
	AUC	75	0.41	73	0.56	2	0.04		ACC	33	0.66	29	0.69	4	0.5
Met	AUA	**260**	**1.7**	**204**	**1.86**	**56**	**1.3**		ACA	**109**	**2.18**	**105**	**2.5**	4	0.5
	AUG	45	0.3	15	0.14	30	0.7		ACG	3	0.06	3	0.07	0	0
Val	GUU	**85**	**2.04**	14	0.7	**71**	**3.26**	Ala	GCU	46	1.38	19	0.81	**27**	**2.77**
	GUC	8	0.19	7	0.35	1	0.05		GCC	32	0.96	26	1.11	6	0.62
	GUA	61	1.46	**53**	**2.65**	8	0.37		GCA	**52**	**1.56**	**49**	**2.09**	3	0.31
	GUG	13	0.31	6	0.3	7	0.32		GCG	3	0.09	0	0	3	0.31
Tyr	UAU	**128**	**1.59**	**41**	**1.17**	**87**	**1.91**	Cys	UGU	39	1.7	6	1	**33**	**1.94**
	UAC	33	0.41	29	0.83	4	0.09		UGC	7	0.3	6	1	1	0.06
TER	UAA	10	2	7	2	3	2	Trp	UGA	**76**	**1.5**	**64**	**1.8**	12	0.8
	UAG	0	0	0	0	0	0		UGG	25	0.5	7	0.2	18	1.2
His	CAU	**43**	**1.21**	**30**	**1.03**	**13**	**2**	Arg	CGU	15	1.13	2	0.23	**13**	**2.89**
	CAC	28	0.79	28	0.97	0	0		CGC	2	0.15	2	0.23	0	0
Gln	CAA	**49**	**1.58**	**45**	**1.91**	4	0.53		CGA	**30**	**2.26**	**28**	**3.2**	2	0.44
	CAG	13	0.42	2	0.09	**11**	**1.47**		CGG	6	0.45	3	0.34	3	0.67
Asn	AAU	**115**	**1.5**	**70**	**1.3**	**45**	**2**	Ser	AGU	26	0.56	5	0.2	21	0.97
	AAC	38	0.5	38	0.7	0	0		AGC	7	0.15	6	0.24	1	0.05
Lys	AAA	**91**	**1.67**	**79**	**1.9**	12	0.92		AGA	**95**	**2.03**	**58**	**2.31**	**37**	**1.71**
	AAG	18	0.33	4	0.1	**14**	**1.08**		AGG	8	0.17	1	0.04	7	0.32
Asp	GAU	**46**	**1.37**	**23**	**1.1**	**23**	**1.84**	Gly	GGU	57	1.03	9	0.28	**48**	**2.09**
	GAC	21	0.63	19	0.9	2	0.16		GGC	13	0.24	7	0.22	6	0.26
Glu	GAA	**68**	**1.58**	**54**	**1.83**	**14**	**1.04**		GGA	**119**	**2.15**	**101**	**3.13**	18	0.78
	GAG	18	0.42	5	0.17	13	0.96		GGG	32	0.58	12	0.37	20	0.87

Table 13 Condon Usage of Pleidae

		N	RSCU	N+	RSCU +	N−	RSCU −			N	RSCU	N+	RSCU +	N−	RSCU −
Phe	UUU	**296**	**1.67**	**122**	**1.49**	**174**	**1.82**	Ser	UCU	**115**	**2.49**	39	1.56	**76**	**3.6**
	UUC	59	0.33	42	0.51	17	0.18		UCC	15	0.33	14	0.56	1	0.05
Leu	UUA	**378**	**4.18**	**207**	**4.14**	**171**	**4.24**		UCA	89	1.93	**69**	**2.76**	20	0.95
	UUG	42	0.46	10	0.2	32	0.79		UCG	8	0.17	4	0.16	4	0.19
	CUU	52	0.58	28	0.56	**24**	**0.6**	Pro	CCU	**70**	**2.24**	**49**	**2.02**	**21**	**3**
	CUC	9	0.1	5	0.1	4	0.1		CCC	20	0.64	16	0.66	4	0.57
	CUA	**54**	**0.6**	**44**	**0.88**	10	0.25		CCA	31	0.99	29	1.2	2	0.29
	CUG	7	0.08	6	0.12	1	0.02		CCG	4	0.13	3	0.12	1	0.14
Ile	AUU	**350**	**1.8**	**225**	**1.74**	**125**	**1.91**	Thr	ACU	**86**	**2.08**	**58**	**1.8**	**28**	**3.11**
	AUC	39	0.2	33	0.26	6	0.09		ACC	14	0.34	13	0.4	1	0.11
Met	AUA	**305**	**1.85**	**225**	**1.93**	**80**	**1.67**		ACA	60	1.45	57	1.77	3	0.33
	AUG	24	0.15	8	0.07	16	0.33		ACG	5	0.12	1	0.03	4	0.44
Val	GUU	**72**	**1.92**	25	1.23	**47**	**2.72**	Ala	GCU	50	1.56	26	1.12	**24**	**2.74**
	GUC	5	0.13	3	0.15	2	0.12		GCC	19	0.59	17	0.73	2	0.23
	GUA	64	1.71	**51**	**2.52**	13	0.75		GCA	**55**	**1.72**	**48**	**2.06**	7	0.8
	GUG	9	0.24	2	0.1	7	0.41		GCG	4	0.12	2	0.09	2	0.23
Tyr	UAU	**141**	**1.75**	**58**	**1.55**	**83**	**1.93**	Cys	UGU	**40**	**1.82**	**9**	**1.64**	**31**	**1.88**
	UAC	20	0.25	17	0.45	3	0.07		UGC	4	0.18	2	0.36	2	0.12
TER	UAA	7	1.56	5	1.67	2	1.33	Trp	UGA	**85**	**1.73**	**63**	**1.88**	**22**	**1.42**
	UAG	2	0.44	1	0.33	1	0.67		UGG	13	0.27	4	0.12	9	0.58
His	CAU	**50**	**1.43**	**36**	**1.33**	**14**	**1.75**	Arg	CGU	24	1.92	10	1.33	**14**	**2.8**
	CAC	20	0.57	18	0.67	2	0.25		CGC	0	0	0	0	0	0
Gln	CAA	**53**	**1.89**	**45**	**2**	**8**	**1.45**		CGA	24	1.92	**19**	**2.53**	5	1
	CAG	3	0.11	0	0	3	0.55		CGG	2	0.16	1	0.13	1	0.2
Asn	AAU	**168**	**1.71**	**105**	**1.6**	**63**	**1.94**	Ser	AGU	42	0.91	17	0.68	25	1.18
	AAC	28	0.29	26	0.4	2	0.06		AGC	6	0.13	2	0.08	4	0.19
Lys	AAA	**97**	**1.67**	**77**	**1.81**	**20**	**1.29**		AGA	**91**	**1.97**	**55**	**2.2**	**36**	**1.7**
	AAG	19	0.33	8	0.19	11	0.71		AGG	3	0.07	0	0	3	0.14
Asp	GAU	**55**	**1.62**	**32**	**1.45**	**23**	**1.92**	Gly	GGU	**97**	**2.01**	38	1.3	**59**	**3.11**
	GAC	13	0.38	12	0.55	1	0.08		GGC	7	0.15	6	0.21	1	0.05
Glu	GAA	**65**	**1.57**	**48**	**1.71**	**17**	**1.26**		GGA	63	1.31	**55**	**1.88**	8	0.42
	GAG	18	0.43	8	0.29	10	0.74		GGG	26	0.54	18	0.62	8	0.42

Table 14 Condon Usage of Reduviidae

		N	RSCU	N+	RSCU +	N−	RSCU −			N	RSCU	N+	RSCU +	N−	RSCU −
Phe	UUU	**292**	**1.68**	**113**	**1.37**	**179**	**1.97**	Ser	UCU	**108**	**2.32**	33	1.24	**75**	**3.77**
	UUC	55	0.32	52	0.63	3	0.03		UCC	28	0.6	25	0.94	3	0.15
Leu	UUA	**323**	**3.68**	**178**	**3.62**	**145**	**3.77**		UCA	101	2.17	**76**	**2.85**	25	1.26
	UUG	52	0.59	6	0.12	46	1.19		UCG	2	0.04	1	0.04	1	0.05
	CUU	**82**	**0.94**	47	0.96	**35**	**0.91**	Pro	CCU	**69**	**2.08**	40	1.58	**29**	**3.62**
	CUC	9	0.1	8	0.16	1	0.03		CCC	19	0.57	17	0.67	2	0.25
	CUA	53	0.6	**50**	**1.02**	3	0.08		CCA	45	1.35	**44**	**1.74**	1	0.12
	CUG	7	0.08	6	0.12	1	0.03		CCG	0	0	0	0	0	0
Ile	AUU	**325**	**1.78**	**216**	**1.68**	**109**	**2**	Thr	ACU	76	1.63	46	1.22	**30**	**3.43**
	AUC	41	0.22	41	0.32	0	0		ACC	16	0.34	16	0.42	0	0
Met	AUA	**250**	**1.78**	**169**	**1.89**	**81**	**1.59**		ACA	**92**	**1.98**	**88**	**2.33**	4	0.46
	AUG	31	0.22	10	0.11	21	0.41		ACG	2	0.04	1	0.03	1	0.11
Val	GUU	**92**	**1.88**	34	1.31	**58**	**2.52**	Ala	GCU	**65**	**1.83**	30	1.22	**35**	**3.18**
	GUC	9	0.18	7	0.27	2	0.09		GCC	15	0.42	14	0.57	1	0.09
	GUA	86	1.76	**62**	**2.38**	24	1.04		GCA	60	1.69	**54**	**2.2**	6	0.55
	GUG	9	0.18	1	0.04	8	0.35		GCG	2	0.06	0	0	2	0.18
Tyr	UAU	**138**	**1.73**	**51**	**1.46**	**87**	**1.93**	Cys	UGU	47	1.65	9	0.95	**38**	**2**
	UAC	22	0.28	19	0.54	3	0.07		UGC	10	0.35	10	1.05	0	0
TER	UAA	6	1.5	3	1.2	3	2	Trp	UGA	**90**	**1.78**	**69**	**1.94**	21	1.4
	UAG	2	0.5	2	0.8	0	0		UGG	11	0.22	2	0.06	9	0.6
His	CAU	**58**	**1.47**	**40**	**1.31**	**18**	**2**	Arg	CGU	19	1.43	5	0.57	**14**	**3.11**
	CAC	21	0.53	21	0.69	0	0		CGC	1	0.08	1	0.11	0	0
Gln	CAA	**57**	**1.84**	**46**	**1.92**	**11**	**1.57**		CGA	**29**	**2.19**	**27**	**3.09**	2	0.44
	CAG	5	0.16	2	0.08	3	0.43		CGG	4	0.3	2	0.23	2	0.44
Asn	AAU	**130**	**1.64**	**81**	**1.5**	**49**	**1.92**	Ser	AGU	28	0.6	9	0.34	19	0.96
	AAC	29	0.36	27	0.5	2	0.08		AGC	10	0.22	9	0.34	1	0.05
Lys	AAA	**69**	**1.48**	**56**	**1.78**	13	0.87		AGA	**91**	**1.96**	**60**	**2.25**	**31**	**1.56**
	AAG	24	0.52	7	0.22	**17**	**1.13**		AGG	4	0.09	0	0	4	0.2
Asp	GAU	**54**	**1.61**	**31**	**1.41**	**23**	**2**	Gly	GGU	67	1.2	18	0.54	**49**	**2.18**
	GAC	13	0.39	13	0.59	0	0		GGC	8	0.14	3	0.09	5	0.22
Glu	GAA	**63**	**1.52**	**51**	**1.82**	12	0.89		GGA	**115**	**2.06**	**98**	**2.95**	17	0.76
	GAG	20	0.48	5	0.18	**15**	**1.11**		GGG	33	0.59	14	0.42	19	0.84

TER, stop codon (single T stop codon was not included in this talbe); RSCU, relative synonymous codon usage; n^*, total number in double strands; $RSCU^*$, RSCU in double strands; n+, total number in the plus strand; RSCU+, RSCU in the plus strand; n-, total number in the minus strand; RSCU-, RSCU in the minus strand.

Nucleotide composition

Among the mt-genomes sequenced in this study, the nucleotide compositions are biased toward adenines and thymines. The nucleotide skew statistics of J-strands indicate that the heteropterans are AT-skewed and CG-skewed. Although several mt-genomes are not completely sequenced, these skew trends are obvious.

Table 15 Nucleotide composition of Aphelocheiridae

	Length(bp)	A%	G%	C%	T%	A+T %	AT-skew	CG-skew
Whole_J	15625	41.4	10.3	15.9	32.4	73.7	0.123	0.213
PCGs	11087	31.2	13.4	13.4	42.0	73.2	-0.148	-0.001
PCGs_J	6832	35.5	12.1	16.5	35.9	71.4	-0.007	0.150
PCGs_N	4255	24.4	15.4	8.5	51.8	76.1	-0.360	-0.291
tRNAs	1450	38.6	13.7	10.4	37.4	75.9	0.015	-0.135
tRNAs_J	921	40.3	12.2	11.6	35.9	76.2	0.057	-0.023
tRNAs_N	529	35.5	16.3	8.3	39.9	75.4	-0.058	-0.323
rRNAs	2033	33.5	15.8	8.5	42.2	75.7	-0.114	-0.300
Control	724	35.9	11.3	18.8	34.0	69.9	0.028	0.248

Table 16 Nucleotide composition of Belostomatidae

	Length(bp)	A%	G%	C%	T%	A+T %	AT-skew	CG-skew
Whole_J	14574	42.6	10.9	14.3	32.1	74.7	0.140	0.134
PCGs	11088	33.0	13.2	12.9	40.9	73.9	-0.106	-0.010
PCGs_J	6812	37.7	12.3	14.8	35.2	72.9	0.035	0.091
PCGs_N	4276	25.6	14.5	9.9	50.0	75.6	-0.323	-0.189
tRNAs	1454	40.2	12.9	9.2	37.8	77.9	0.031	-0.165
tRNAs_J	927	42.0	12.2	10.5	35.4	77.3	0.085	-0.076
tRNAs_N	527	37.0	14.0	7.0	41.9	78.9	-0.063	-0.333
rRNAs	2040	33.2	14.0	9.0	43.9	77.1	-0.139	-0.218
Control								

Table 17 Nucleotide composition of Corixidae

	Length(bp)	A%	G%	C%	T%	A+T %	AT-skew	CG-skew
Whole_J	14596	40.9	12.5	17.7	28.9	69.8	0.173	0.172
PCGs	11104	29.4	15.5	16.1	39.0	68.3	-0.140	0.019
PCGs_J	6829	34.2	14.5	19.4	32.0	66.2	0.034	0.146
PCGs_N	4275	21.7	17.2	10.9	50.2	71.8	-0.396	-0.224
tRNAs	1418	36.8	14.7	10.4	38.0	74.8	-0.016	-0.171
tRNAs_J	915	39.5	13.3	11.0	36.2	75.6	0.043	-0.094
tRNAs_N	525	32.6	17.7	9.5	40.2	72.8	-0.105	-0.301
rRNAs	2051	29.6	15.8	9.7	44.9	74.5	-0.205	-0.240
Control								

Table 18 Nucleotide composition of Fulgoridae

	Length(bp)	A%	G%	C%	T%	A+T %	AT-skew	CG-skew
Whole_J	15724	41.3	10.5	14.3	33.8	75.2	0.100	0.154
PCGs	11043	33.8	12.8	12.5	41.0	74.8	-0.096	-0.012
PCGs_J	6774	37.5	12.0	14.3	36.2	73.6	0.018	0.087
PCGs_N	4269	28.0	13.9	9.4	48.7	76.7	-0.270	-0.190
tRNAs	1442	39.3	13.4	9.4	38.0	77.3	0.016	-0.177
tRNAs_J	917	40.0	13.2	10.8	36.0	76.0	0.053	-0.100
tRNAs_N	525	37.9	13.7	6.9	41.5	79.4	-0.046	-0.333
rRNAs	2019	33.7	14.0	8.5	43.8	77.6	-0.130	-0.245
Control	772	42.7	8.9	14.4	33.9	76.7	0.115	0.233

Table 19 Nucleotide composition of Gelastocoridae

	Length(bp)	A%	G%	C%	T%	A+T %	AT-skew	CG-skew
Whole_J	15410	48.7	8.5	15.2	27.6	76.3	0.277	0.282
PCGs	10959	33.6	11.3	13.4	41.7	75.3	-0.107	0.087
PCGs_J	6738	42.7	9.3	17.2	30.8	73.5	0.163	0.299
PCGs_N	4221	19.1	14.4	7.3	59.2	78.3	-0.511	-0.328
tRNAs	1409	40.5	13.0	10.6	35.8	76.4	0.061	-0.099
tRNAs_J	902	45.7	11.2	11.6	31.5	77.2	0.184	0.019
tRNAs_N	507	31.4	16.2	8.9	43.6	75.0	-0.163	-0.291
rRNAs	2004	28.1	14.2	8.1	49.6	77.7	-0.276	-0.274
Control	1043	47.9	6.8	9.9	35.4	83.3	0.151	0.184

Table 20 Nucleotide composition of Gerridae

	Length(bp)	A%	G%	C%	T%	A+T %	AT-skew	CG-skew
Whole_J	16079	43.1	9.7	16.1	31.0	74.2	0.163	0.247
PCGs	11125	33.0	13.1	12.6	41.3	74.3	-0.111	-0.020
PCGs_J	6832	38.4	11.8	15.3	34.5	72.9	0.054	0.126
PCGs_N	4293	24.4	15.2	8.4	52.0	76.4	-0.362	-0.285
tRNAs	1481	39.6	12.8	9.3	38.4	77.9	0.016	-0.162
tRNAs_J	939	41.3	11.7	10.8	36.2	77.5	0.066	-0.043
tRNAs_N	542	36.5	14.8	6.6	42.1	78.6	-0.070	-0.379
rRNAs	2025	30.0	14.4	8.2	47.4	77.3	-0.225	-0.272
Control	1450	34.6	5.7	29.0	30.8	65.4	0.057	0.673

Table 21 Nucleotide composition of Hydrometridae

	Length(bp)	A%	G%	C%	T%	A+T %	AT-skew	CG-skew
Whole_J	15380	43.9	10.0	14.4	31.8	75.7	0.160	0.179
PCGs	11113	31.3	13.7	14.8	40.2	71.5	-0.124	0.038
PCGs_J	6852	37.2	11.7	18.5	32.6	69.8	0.067	0.225
PCGs_N	4261	21.8	16.9	8.8	52.4	74.2	-0.413	-0.314
tRNAs	1462	39.3	12.9	9.4	38.5	77.8	0.010	-0.157
tRNAs_J	933	42.1	11.6	10.0	36.3	78.5	0.074	-0.075
tRNAs_N	529	34.2	15.1	8.3	42.3	76.6	-0.106	-0.290
rRNAs	2041	30.8	13.4	8.5	47.3	78.1	-0.211	-0.226
Control	781	30.2	7.4	26.4	36.0	66.2	-0.087	0.561

Table 22 Nucleotide composition of Leptopodidae

	Length(bp)	A%	G%	C%	T%	A+T %	AT-skew	CG-skew
Whole_J	15416	43.9	9.0	12.3	34.7	78.7	0.117	0.157
PCGs	11003	36.2	10.7	11.3	41.9	78.1	-0.073	0.027
PCGs_J	6772	40.2	10.3	13.2	36.4	76.6	0.050	0.123
PCGs_N	4231	29.8	11.3	8.2	50.7	80.5	-0.259	-0.156
tRNAs	1431	39.2	11.9	8.5	40.5	79.7	-0.016	-0.168
tRNAs_J	913	40.3	11.1	9.7	38.9	79.2	0.018	-0.063
tRNAs_N	518	37.3	13.3	6.2	43.2	80.5	-0.074	-0.366
rRNAs	2044	34.6	12.0	7.3	46.0	80.7	-0.141	-0.246
Control	694	35.4	7.9	14.0	42.7	78.1	-0.092	0.276

Table 23 Nucleotide composition of Naucoridae

	Length(bp)	A%	G%	C%	T%	A+T %	AT-skew	CG-skew
Whole_J	14516	44.7	10.8	16.8	27.7	72.4	0.236	0.217
PCGs	11062	33.1	13.6	14.5	38.9	71.9	-0.081	0.031
PCGs_J	6808	40.3	12.0	17.8	29.8	70.2	0.149	0.194
PCGs_N	4254	21.4	16.1	9.1	53.4	74.8	-0.427	-0.278
tRNAs	1443	38.0	15.0	10.7	36.2	74.2	0.025	-0.167
tRNAs_J	912	40.8	13.5	12.3	33.4	74.2	0.099	-0.047
tRNAs_N	531	33.3	17.7	8.1	40.9	74.2	-0.102	-0.372
rRNAs	2027	29.6	16.5	9.7	44.2	73.8	-0.199	-0.259
Control								

Table 24 Nucleotide composition of Nepidae

	Length(bp)	A%	G%	C%	T%	A+T %	AT-skew	CG-skew
Whole_J	15209	41.7	12.1	17.0	29.3	71.0	0.175	0.170
PCGs	11121	31.4	14.6	15.2	38.8	70.2	-0.105	0.018
PCGs_J	6834	36.9	13.9	17.9	31.4	68.2	0.080	0.125
PCGs_N	4287	22.7	15.8	10.9	50.5	73.2	-0.379	-0.184
tRNAs	1451	38.3	14.7	10.8	36.1	74.4	0.030	-0.154
tRNAs_J	926	40.4	13.8	11.9	33.9	74.3	0.087	-0.076
tRNAs_N	525	34.7	16.4	9.0	40.0	74.7	-0.071	-0.293
rRNAs	2039	30.2	16.0	9.8	44.0	74.3	-0.186	-0.242
Control	609	28.9	7.7	26.3	37.1	66.0	-0.124	0.546

Table 25 Nucleotide composition of Notonectidae

	Length(bp)	A%	G%	C%	T%	A+T %	AT-skew	CG-skew
Whole_J	15321	43.0	11.2	18.2	27.6	70.6	0.218	0.237
PCGs	11095	30.7	14.7	15.2	39.4	70.1	-0.123	0.018
PCGs_J	6820	37.4	13.0	18.6	31.0	68.4	0.094	0.177
PCGs_N	4275	20.1	17.3	9.8	52.7	72.8	-0.448	-0.276
tRNAs	1436	36.8	15.7	11.1	36.3	73.1	0.008	-0.171
tRNAs_J	917	39.7	13.6	12.9	33.8	73.5	0.080	-0.029
tRNAs_N	519	31.8	19.5	8.1	40.7	72.4	-0.122	-0.413
rRNAs	2044	26.1	16.8	9.5	47.6	73.7	-0.291	-0.279
Control	751	32.0	6.4	27.8	33.8	65.8	-0.028	0.626

Table 26 Nucleotide composition of Ochteridae

	Length(bp)	A%	G%	C%	T%	A+T %	AT-skew	CG-skew
Whole_J	15262	44.4	9.6	14.3	31.7	76.1	0.166	0.199
PCGs	11121	34.6	12.0	12.2	41.2	75.8	-0.088	0.011
PCGs_J	6834	40.1	10.9	14.4	34.5	74.7	0.075	0.140
PCGs_N	4287	25.7	13.7	8.7	51.9	77.6	-0.338	-0.220
tRNAs	1474	40.2	12.6	9.4	37.9	78.0	0.030	-0.148
tRNAs_J	938	42.9	11.0	10.2	35.9	78.8	0.088	-0.035
tRNAs_N	536	35.4	15.5	7.8	41.2	76.7	-0.075	-0.328
rRNAs	2042	31.6	13.8	8.1	46.5	78.2	-0.190	-0.260
Control	646	36.4	5.4	24.1	34.1	70.4	0.033	0.634

Table 27 Nucleotide composition of Pleidae

	Length(bp)	A%	G%	C%	T%	A+T %	AT-skew	CG-skew
Whole_J	14609	43.6	10.2	17.1	29.2	72.7	0.198	0.251
PCGs	11113	31.3	13.7	14.8	40.2	71.5	-0.124	0.038
PCGs_J	6852	37.2	11.7	18.5	32.6	69.8	0.067	0.225
PCGs_N	4261	21.8	16.9	8.8	52.4	74.2	-0.413	-0.314
tRNAs	1456	39.4	14.1	9.3	37.2	76.6	0.028	-0.202
tRNAs_J	927	42.2	12.5	10.7	34.6	76.8	0.098	-0.079
tRNAs_N	529	34.4	16.8	7.0	41.8	76.2	-0.097	-0.413
rRNAs	2032	29.6	15.5	7.9	47.0	76.6	-0.228	-0.324
Control								

Table 28 Nucleotide composition of Reduviidae

	Length(bp)	A%	G%	C%	T%	A+T %	AT-skew	CG-skew
Whole_J	15130	42.4	9.7	13.9	34.1	76.5	0.108	0.178
PCGs	11092	32.6	12.1	12.1	43.2	75.8	-0.140	0.003
PCGs_J	6831	36.6	11.2	14.3	37.8	74.4	-0.016	0.123
PCGs_N	4261	26.1	13.4	8.6	51.8	78.0	-0.330	-0.220
tRNAs	1439	38.8	12.0	9.7	39.5	78.2	-0.009	-0.105
tRNAs_J	920	41.0	11.1	11.1	36.8	77.8	0.053	0.000
tRNAs_N	519	34.9	13.7	7.3	44.1	79.0	-0.117	-0.303
rRNAs	2028	33.8	12.8	7.9	45.5	79.3	-0.147	-0.233
Control	608	30.9	6.7	18.3	44.1	75.0	-0.175	0.461

References

1. Lynch M: **Mutation accumulation in nuclear, organelle, and prokaryotic transfer RNA genes.** *Mol Biol Evol* 1997, **14:**914-925.
2. Tsaousis AD, Martin DP, Ladoukakis ED, Posada D, Zouros E: **Widespread recombination in published animal mtDNA sequences.** *Mol Biol Evol* 2005, **22:**925-933.

3. Adams KL, Palmer JD: **Evolution of mitochondrial gene content: gene loss and transfer to the nucleus.** *Mol Phylogenet Evol* 2003, **29:**380-395.
4. Podsiadlowski L, Braband A: **The complete mitochondrial genome of the sea spider *Nymphon gracile* (Arthropoda: Pycnogonida).** *BMC Genomics* 2006, **7:**284.
5. Lavrov DV, Brown WM, Boore JL: **A novel type of RNA editing occurs in the mitochondrial tRNAs of the centipede *Lithobius forficatus*.** *Proc Natl Acad Sci U S A* 2000, **97:**13738-13742.
