# Supplementary material for: Phylogenetic analysis of the true water bugs (Insecta: Hemiptera: Heteroptera: Nepomorpha): evidence from mitochondrial genomes
Source: BMC Evol Biol. 2009 Jun 15;9:134. doi: 10.1186/1471-2148-9-134 (PMC2711072; doi:10.1186/1471-2148-9-134)
Supplement: Additional file 3 — Nucleotide substitution saturation analysis. The data provided represent the nucleotide substitution saturation analysis of different portions of datasets. [file 1471-2148-9-134-S3.pdf]

Nucleotide substitution saturation analysis

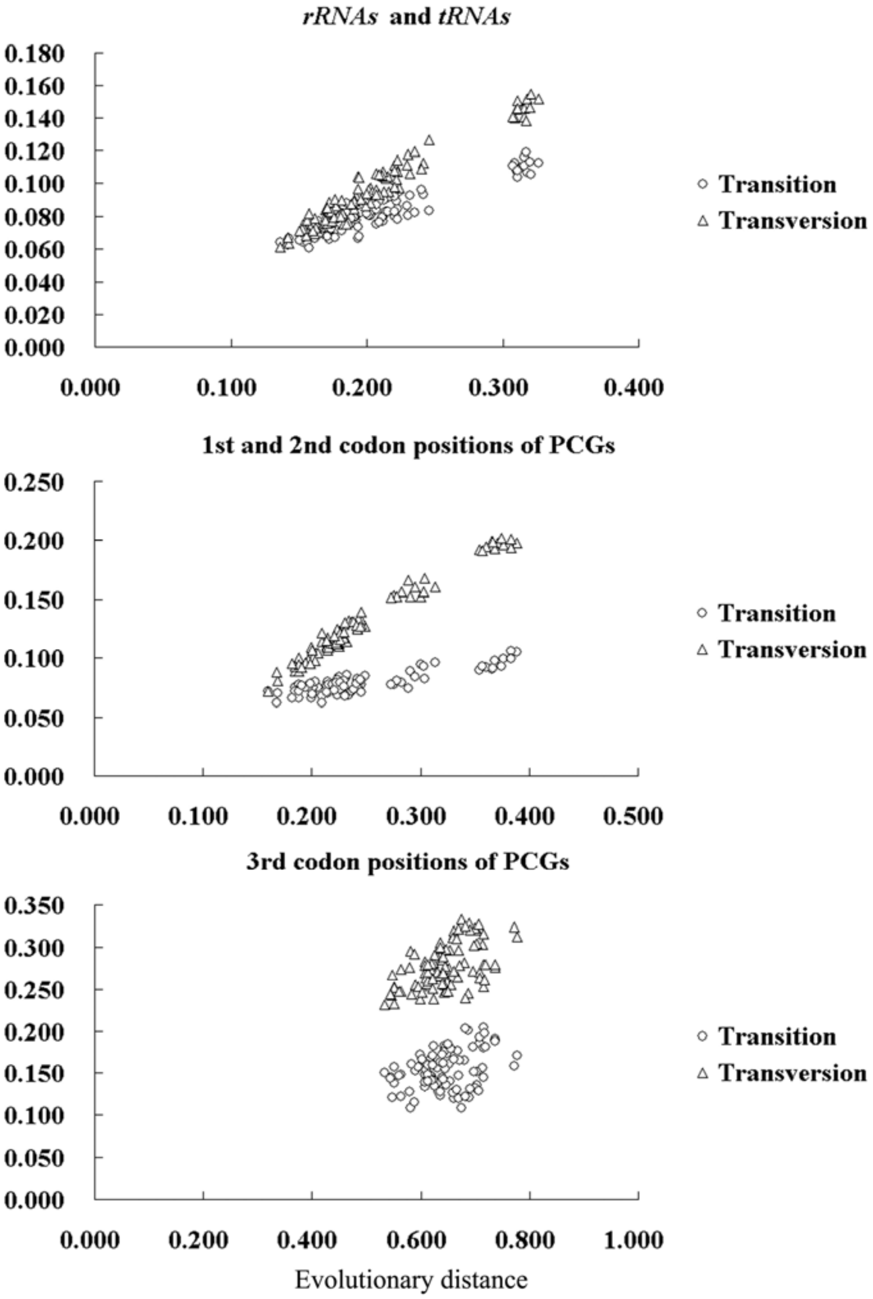

Figure 1. Nucleotide substitution pattern of different portions of datasets used in phylogenetic analysis. Transition and transversion substitutions per site were poltted against pairwise evolutionary distances (K80 model).

Both the pairwise transition and transversiona differences for RT (*rRNA*s and *tRNAs*) and PCG12 (the first and second codon positions of PGCs) increased with increasing evolutionary distance, with the exception of the third codon positions of the PCGs (Figure 1), in which glaring saturation could be observed. It was apparent that some degree of saturation also occurred in PCG12 dataset particularly transitions, although pairwise differences seemed to accumulate steadily along the X axis. Then

in phylogenetic analysis, the third codon positions are troublesome and some noise are brought by transitions of PCG12 dataset. Therefore, we don’t think that the 3 trees derived from the suboptimal datasets (Bayesian PCG123 tree, ML PCG123 tree, and ML PCG12 tree) would affect the final conclusions because the majority of the analyses are consistent.
