# Supplementary figures and images for: Phylogenetic analysis of the true water bugs (Insecta: Hemiptera: Heteroptera: Nepomorpha): evidence from mitochondrial genomes
Source: BMC Evol Biol. 2009 Jun 15;9:134. doi: 10.1186/1471-2148-9-134 (PMC2711072; doi:10.1186/1471-2148-9-134)

Bayesian trees

PCG123RT

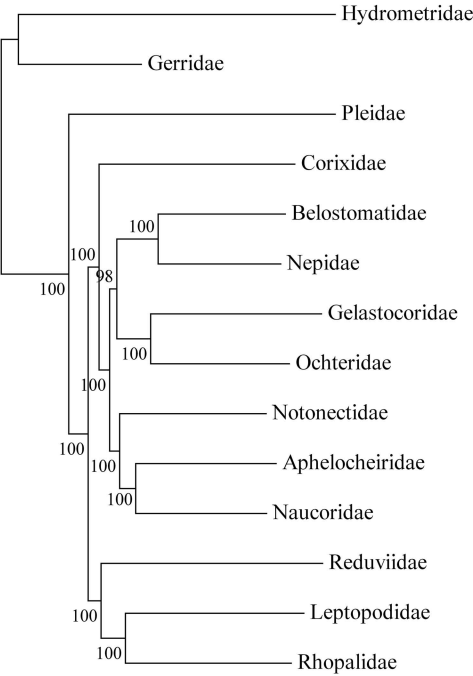

0.1

PCG12RT

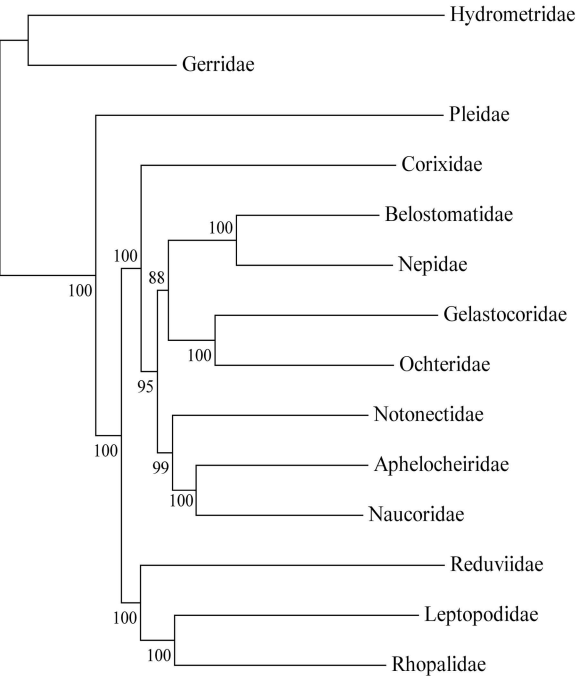

0.1

PCG123

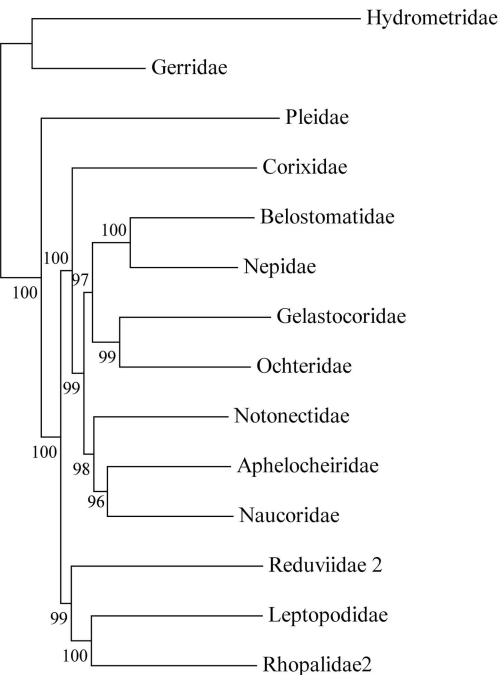

0.1

PCG12

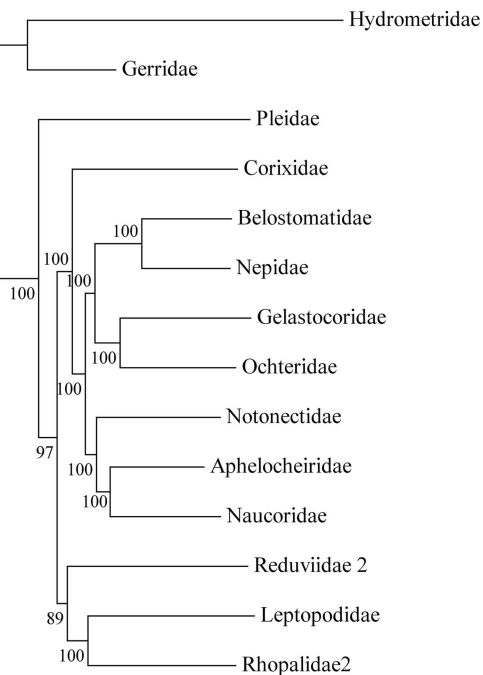

0.1

# ML trees

PCG123RT

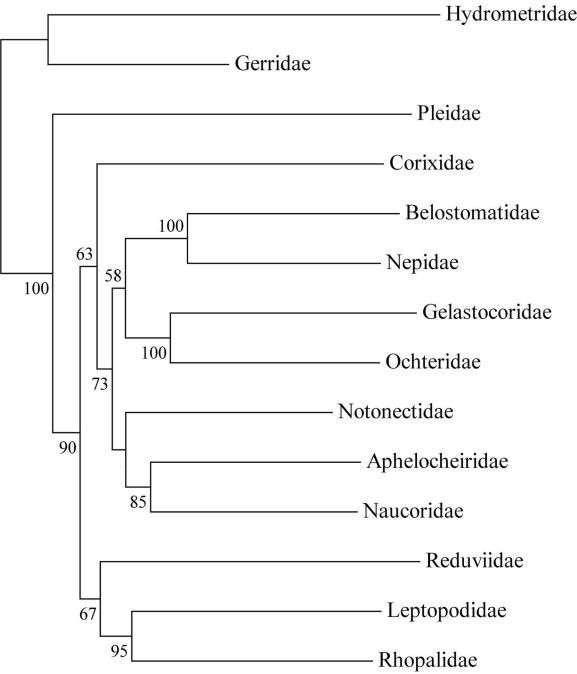

PCG12RT

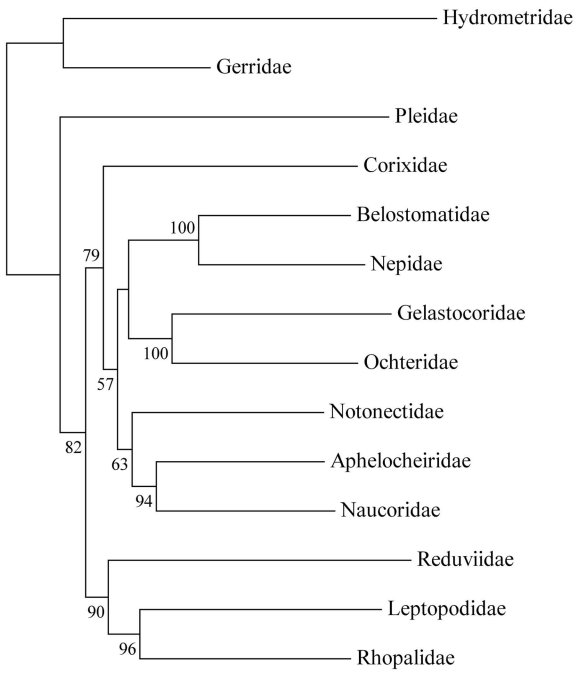

PCG123

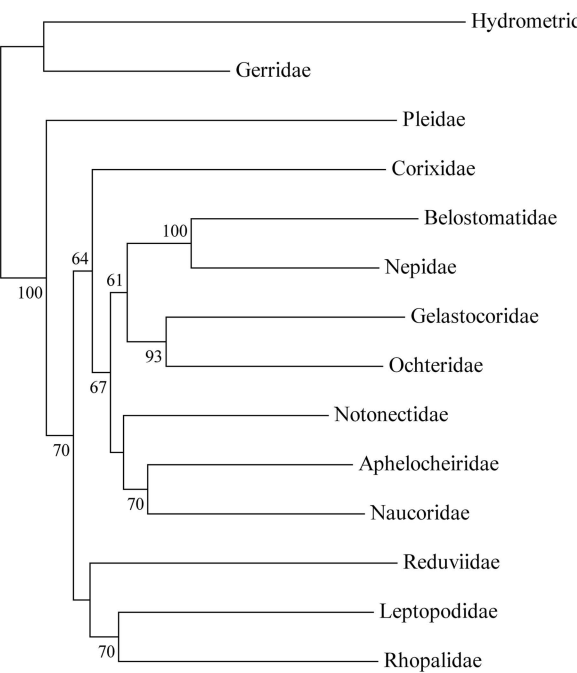

PCG12

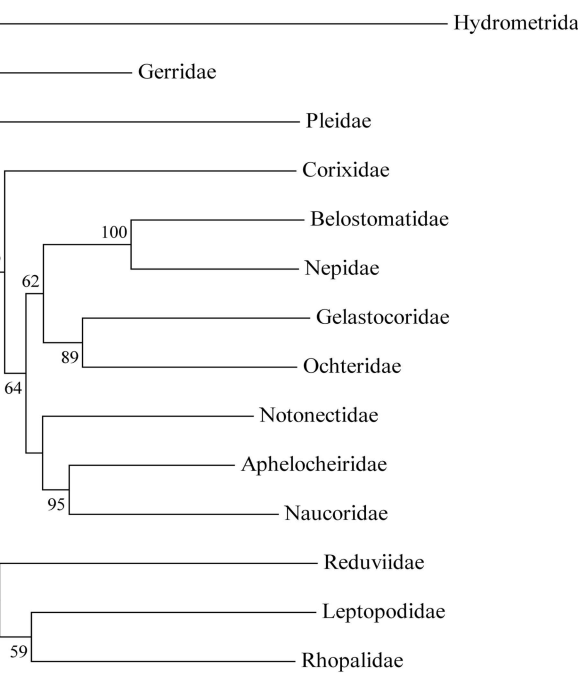

Supplement: Additional file 4 — Bayesian and ML phylograms inferred from the data sets without Lycorma delicatula (Insecta: Hemiptera: Archaeorrhyncha). The data provided represent Bayesian and ML phylograms inferred from the data sets without Lycorma delicatula (Insecta: Hemiptera: Archaeorrhyncha). [file 1471-2148-9-134-S4.pdf]
