# Supplementary material for: Phylogenetic analysis of the true water bugs (Insecta: Hemiptera: Heteroptera: Nepomorpha): evidence from mitochondrial genomes
Source: BMC Evol Biol. 2009 Jun 15;9:134. doi: 10.1186/1471-2148-9-134 (PMC2711072; doi:10.1186/1471-2148-9-134)
Supplement: Additional file 5 — Primers used in PCR. The data provided represent primers used in amplification of mt-genomes. [file 1471-2148-9-134-S5.pdf]

Supplementary material 1. PCR primers used in this study.

**Primer**	**Sequence 5'- 3'**
CO1F[a]	GGAACAGGATGAACAGTTTACCCTCC
CO1R[b]	TCTGAATATCGTCGAGGTATTCC
CytbF[a]	TATGTTCTTCCCTGAGGACAAATATC
CytbR[a]	ATTACACCTCCTAATTTATTAGGAAT
Hyd.CO1F	TCATCACATATTTACCGTAGGAATAGATG
Hyd.CytbR	TTCCTGATGGATTGTTTGACCCAGTTAT
Hyd.CytbF	TAACTGGGTCAAACAATCCATCAGGAACA
Hyd.CO1R	TCAACATCTATTCCTACGGTAAATATGTGA
Ger.CO1F	GTAGATACACGAGCATACTTTACATCAGCA
Ger.CytbR	GCAAGAATAAATGGAAGTAAGAAATGTAGT
Ger.CytbF	TCCTACTGGTATTAACAGAAATTATGACA
Ger.CO1R	GAATAGGTGTTGATAAAGTACGGGGTCC
Red.CO1F	TCCTGTTCACTATTGGAGGTTTAACGGGT
Red.CytbR	GCTAAGACGAAGGGTAAAAGAAAGTGTA
Red.CytbF	TTACACTTTCTTTTACCCTTCGTCTTAG
Red.CO1R	CGTTCAGGTGTTATACCATTAGGTCGTA
Lep.CO1F	GGTATTAAGGTATTCAGATGATTAGCCA
Lep.CytbR	CCTAAGATAATGAACGGTAATAAGAAATGA
Lep.CytbF	CCCCACAGGATTAAATAGAAACTTTGACA
Lep.CO1R	TGGGTGTCCAAAGAATCAAAATAAGTGT
Ple.CO1F	CCTTCTACTTTATGAGCACTGGGTTTTGTA
Ple.CytbR	AGGGGGTTAGACGAGCCAGTTTGATGTA
Ple.CytbF	TACCTTCCCGCCAATCCTCTAGTTACA
Ple.CO1R	CTCCCGCTGGGTCAAAGAAAGATGTA
Not.CO1F	CTTTAGATGAATAGCAACTTTACACGGGAC
Not.CytbR	GATCTCCTAGTATTCGAGGTTCTAGTAAGT
Not.CytbF	GCAACACTAACTCGATTCTTTACATTACACTT
Not.CO1R	CTGTCCCGTGTAAAGTTGCTATTCATCTAA
Och.CO1F	TATGTAGTTGCTCATTTCCATTACG
Och.CytbR	AGAAGTATGGGTGGAAGGGGATTTTGTCA
Och.CytbF	TAATAAATCTCCTTGAGCCACGCCTTCT
Och.CO1R	CTGGTAGTGATAGAAGTAATAACAGGGCT
Gel.CO1F	TTTACAGTAGGTATAGATGTGGATACACGAG
Gel.CytbR	GGAAGTATCATTCTGGTTGAATATGTACTGG
Gel.CytbF	AGCAATCCCGTATCTAGGACCTGAAATAG
Gel.CO1R	AGCCATTCATCTAAATACTTTAATACCTGTAG
Nep.CO1F	CCCTCCTATTACTCCTATCGCTACCC
Nep.CytbR	TCCTAGCGGGTTATTAGATCCTGTTTGATG
Nep.CytbF	CCGTTTCACCCATACTTTACGACAAAGGA
Nep.CO1R	TAGCGATAGGAGTAATAGGAGGGCGGTA

Bel.CO1F	AGCCACTTTATGGGCATTAGGATTTGTA
Bel.CytbR	GCGATTACGAAGGGCAATAAAAAGTGTA
Bel.CytbF	ATATTGATAAAGTGCCGTTTCACCCATAC
Bel.CO1R	ATTTTTACCTCTTTCTTGGCTAATGATGTG
Cor.CO1F	ATAGGAGCAGTATTTGCGATTATTGGTAGA
Cor.CytbR	GGTAGTAGAAAATGTAAAGTGAAAAATCGTGT
Cor.CytbF	CACGATTTTTCACTTTACATTTTCTACTACCA
Cor.CO1R	TAGTACCGTGAAGAGTTGCTAATCATCTGA
Aph.CO1F	CAACACTTATTTTGATTTTTTGGTCACCCA
Aph.CytbR	GGTAGTAGGAAATGTAAAGTGAAGAATC
Aph.CytbF	GATTCTTCACTTTACATTTCCTACTACCA
Aph.CO1R	AATGCTTCATTTTTTCCTCTTTCTTGTCTA
Nau.CO1F	GTAGATACACGAGCATACTTTACATCAGCA
Nau.CytbR	ATCCTGTTTGGTGTAGAAATAGCAAGTGAA
Nau.CytbF	ACGATTCTTTACTTTACATTTTATCCTGCCA
Nau.CO1R	TTATGGTTGCTGATGTAAAGTATGCTCGTG
Ful.CytbF	ACAATGAGTATGAGGAGGATTCGCTGTAGA
Ful.CO1R	GCTCGTGTGTCAATATCTATACCTACAGTA
Ful.CO1F	ACTGTAGGTATAGATATTGACACACGAGCA
Ful.CytbR	GGGTGTTCTTAGTGGGTTTGCTGGAGTA

[a] were modified from (Simon et al., 1994). [b] was designed from the comparisons of other insects. Other primers were designed from the sequenced fragments of *CO1* and *Cytb* amplified with CO1F & CO1R and CytbF & CytbR. Each pair of primers special for each species to amplify two long fragments were named with the first three letters of the superfamily / family name in order to be intuitionistic.
